# An ensemble heterogeneous transformer model for an effective diagnosis of multiple plant diseases

**DOI:** 10.3389/fpls.2025.1693095

**Published:** 2025-12-03

**Authors:** Ponugoti Kalpana, Pradeepini Gera, Eatedal Alabdulkreem, Mohammad Tabrez Quasim, Jamel Baili, Yongwon Cho, Yunyoung Nam

**Affiliations:** 1 Department of Computer Science and Engineering, AVN Institute of Engineering and Technology, Hyderabad, Telangana, India; 2 Department of Computer Science and Engineering, Koneru Lakshmaiah University, Vijayawada, Andhra Pradesh, India; 3 Department of Computer Sciences, College of Computer and Information Sciences, Princess Nourah bint Abdulrahman University, Riyadh, Saudi Arabia; 4 Department of Computer Science and Artificial Intelligence, College of Computing and Information Technology, University of Bisha, Bisha, Saudi Arabia; 5 Department of Computer Engineering, College of Computer Science, King Khalid University, Abha, Saudi Arabia; 6 Department of Computer Science and Engineering, Soonchunhyang University, Asan, Republic of Korea

**Keywords:** CoATNets, ensemble deep learning architectures, plant diseases, plant village datasets, transformer neural networks

## Abstract

Plant diseases are a significant challenge to sustainable farming resulting in drastic losses of crop quality and quantity. Conventional diagnostic procedures like manual examination and single-model deep learning-based methods tend to be ineffective in identifying overlapping appearances, detailed textures of leaves, and environmental changes, which results in inconsistent performance. In order to address these issues, this paper presents an ensemble transformer framework that incorporates the segmentation, classification and optimization to identify multi-diseases in plants accurately. The framework has a two phase design. At the initial stage, U-Net and Swin Transformer V2 detect the disease-affected leaf areas with high accuracy, and the important features are correctly captured. In the second stage, classification is carried out using CoAtNet and its enhanced variant, which combine convolutional feature extraction with transformer-based global context learning. To further improve decision-making, a meta-heuristic fusion strategy based on the Levy Flight Honey Badger Algorithm dynamically weights classifier outputs, enhancing robustness and reducing misclassifications. Model interpretability is enhanced through GRAD-CAM visualizations, providing clear insights into the regions influencing disease classification. The framework was extensively evaluated on the PlantVillage dataset containing 54,305 images across 38 classes. Results demonstrate outstanding performance, with 99.31% accuracy, 99.32% precision, 99.31% recall, 99.32% specificity, and 99.31% F1-score. The ensemble segmentation approach exhibits a statistically significant 7.34% improvement over single-method implementations. Moreover, the heterogeneous ensemble model achieves 8.43% and 14.59% superiority over homogeneous ensembles and individual models, respectively. The integration of segmentation, hybrid transformer architectures, and meta-heuristic decision fusion delivers a powerful, interpretable, and highly reliable solution for early plant disease detection, offering strong potential for real-world agricultural deployment.

## 1 Introduction

Agriculture remains a cornerstone of global food security, yet plant diseases cause severe damage to crop production and food production. These diseases, often caused by bacterial, fungal, or viral pathogens, can lead to substantial economic losses if not identified and treated promptly (Ferentinos, 2021; Mohanty et al., 2021; Picon et al., 2021; Too et al., 2021). Traditional methods for detecting plant diseases rely heavily on hand inspection by specialists, a process that is both time-consuming and prone to human error. The lack of advanced diagnostic tools in many agricultural regions further exacerbates this issue, leading to delayed intervention and increased crop losses (Arsenovic et al., 2021). Timely and accurate diagnosis of plant diseases is notable in the cases of effective governance and risk minimization. Nevertheless, traditional models based on images such as Convolutional Neural Networks (CNNs) tend to fail with the involvement of complicated patterns of leaves, lighting cycle changes, and the emergence of diseased symptoms overlapping with each other (Duhan et al., 2024; Mishra et al., 2025). Also, single-model solutions are not robust and may not be reliable in the application of large volumes of data in agriculture (Rangarajan et al., 2021). On these issues, the integration of modern DL processes, especially Transformer Neural Networks (TNNs), has been acknowledged as a good way to improve the accuracy of the resulting disease classification process.

A wide variety of Machine Learning and DL technologies have been used to perform effective diagnosis of various plant diseases. The superior image processing operations plays an imperative role in preprocessing the images to boost feature identification and segregation. The feature extraction using Convolutional Neural Networks (CNNs) have also been applied because it is able to capture spatial hierarchies in the plant images (Sibiya and Sumbwanyambe, 2021). Recurrent Neural Networks (RNNs) as well as the Long Short-Term Memory (LSTM) networks have been utilized to perform analysis on sequential data and this has been the case especially when using time-series-based disease pattern recognition. Self-attention mechanisms using transformers, which is the new paradigm in deep learning, have given considerable enhancement in plant diseases classification. Hybrid CNN-transformer models allow learning features that can take advantage of the local and global data in plant images (Chen et al., 2021).

Transfer learning approaches have made it possible to adapt the ready-trained models to agricultural data and thus increasing the classification accuracy with less reliance on numerous training data. Also, ensemble learning mechanisms such as heterogeneous ensemble has been exploited to introduce robustness and generalization through combination of various model architectures (Wang et al., 2025). H parameter optimization Hyperparameters determined by an optimization algorithm, like genetic algorithms and particle swarm optimization, can be used to optimize them towards better performance of the model. Decision fusion strategies that are metaheuristic-based have also been integrated to improve the classification performance through combining predictions taken with a variety of DL methods.

Leaf textures of plants and their obscure disease symptoms make it even more difficult to classify accurately. A lot of plant diseases visually share some common properties and will be incorrectly identified when simple, surface-level models are used. Moreover, the co-occurring disease infections in plants create even more trouble in detecting since it requires multi-label classification methods that are highly complex (Li et al., 2021; Zhou and Zhang, 2021). The other issue is having little access to high-quality and annotated datasets. Such available datasets are biased in the number of samples such that some diseases of plants only have few samples by comparison to others. This imbalance leads to the skewed predictions on the models which in turn influence models classification performance. The method of data augmentation has been tried in this regard, but it has limited success because of so highly imbalanced data sets. The other major issue that makes it difficult to use deep learning models in large-scale applications in agriculture is computational complexity. The time and resources needed in training deep neural networks are enormous and thus, it cannot work on small-scale farmers because most of them have minimum access to high-performance devices. The innovative approaches to model compression, including pruning and quantization have been tested to minimize model size and speed of inference, but their effect on the accuracy should be optimized carefully.

The difficulty with deployment is the combination of deep learning models to current agricultural infrastructure. A large population of farmers and other agricultural specialists are unexperienced in the technical aspect of AI-based systems, which is a technical barrier in the implementation of automated disease detection systems (Liu et al., 2021). There should be training and educational programs to fill this gap and facilitated the practical implementation of AI-derived diagnostic platforms in agriculture.

### 1.1 Problem formulation

A number of issues constrain the performance of conventional methods of detecting plant diseases. Among the most urgent problems, we can distinguish a wide range of species of the plant, environmental conditions and symptoms of the disease so that it is rather challenging to create a common model of the disease detecting. Moreover, a significant proportion of available datasets are insufficiently large, becomes unbalanced/unrepresentative, and therefore reproduces models that fail to generalize on diverse situations (Khan et al., 2021). The variety of the texture and pattern increase the complexity of plant leaf images making the distinction of healthy and diseased areas further complicated in deep learning. These factors usually lead to high false positive and false negative results and narrow the chance of diagnosing diseases early enough. The second challenge is general complexity of computation needed to train DL methods on large agricultural data (Zhang et al., 2021). The processes of training can be lengthy, especially in the case when the structures demanding a lot of resources and hyperparameter tuning are used. Moreover, the decision fusion and optimization is also more complicated with an array of models that need to be integrated in an ensemble framework (Ferentinos, 2018). This calls on the research to seek the deployment of superior methods like ensemble learning and meta-heuristic optimization to enhance better model performance and an increase in the prediction accuracy.

### 1.2 Motivation of the study

The main justification of the present study is the growing need in effective and accurate systems of plant diseases diagnostics which plays a crucial role in overcoming the existing challenges in agriculture caused by the abundance of different plant pathologies. The conventional approaches often lack high rates of accuracy in practice due to the inconsistency in the nature of data and the complexity of symptomatic nature of diseases, no less than to the complexity of the procedures. There is also a need to have a powerful tool to handle large heterogeneous data and also provide proper demarcation of diseased areas. There is a promising avenue of addressing these problems at the price of introducing TNNs, with CoaTNET and Swin Transformers among the early examples of them being effective in global features extraction as well as local features extraction.

The rationale behind the research is mainly to create an ensemble model-based study that synthesizes seamlessly the state-of-the-art image segmentation methodologies and TNNs in the bid to improve plant disease detection. The combination of U-Net in segmentation and CoaTNET/Swin Transformers in classification is a strategic process that can attract the synergistic advantage of two approaches. The objective of this research is to develop a strong, dependable and scalable solution that would be able to contribute to the reduction of crop losses to a significant level and also improve food security on the global level.

### 1.3 Contribution to the field

The recommended approach is a novel generation of plant disease recognition by an ensemble of deep learning models, the combination of advanced image segmentation and the Transformer architecture. The key contributions of the study can be summarized in the following way:

1. Incorporating Segmentation and Transformer Architectures: The concept of the developed framework suggests a two-step solution, in which, U-Net will be used together with Swin Transformer V2 to do image segmentation and CoaTNET, and its Enhanced version to do the labeling of the image segmentation outcomes. Through this integration, accurate locating of the disease affected spots becomes easier before the appropriate classification of the location into the predefined classes is done.
2. Heterogeneous Ensemble Learning: The study contrasts with the conventional homogeneous ensemble methods in the fact that it uses different Transformer models each with its own values to contribute towards. This kind of heterogeneous ensemble design enhances the performance properties of a model that has superior generalization, and toughness which makes it particularly suitable to large and heterogeneous agricultural data.
3. Meta-Heuristic Decision Fusion: It introduces a new meta-heuristic decision fusion and it entails the combination of the output of the remaining ensemble models. This has a benefit of maximizing the final classification accuracy through optimization of the decision process thus better than the single model or homogeneous ensembling approach.
4. Experimental Assessment: The suggested approach is tested using the PlantVillage dataset, which is a collection of 50,000 images of eight disease types. As the results have demonstrated, the strategy is a considerable contribution to Precision, recall, accuracy and F1-score which represents the effectiveness of the tool in early detection of plant diseases.
5. Applicability in Practice: It is noticeable that the model has already been demonstrated to work in a large scale testing and, therefore, could be implemented in the real world agriculture setting, which would not only provide farmers and residents in agrarian areas with a powerful means of early disease detection and prevention, but would also help to avoid the minimal economic loss and enhance the health of crops.

### 1.4 Paper organization

The rest of this paper is organized in such a way that it presents a those-beats overview of the research carried out. Section 2 presents an overview of the available literature related to plant disease detection and the process of optimization, which is used as the basis of the knowledge of the present research. In Section 3, the research gets into the detail of dataset used in the research, discusses the preprocessing steps, transformer neural network model and optimization models. The section 4 describes the implementation details, experiment design, and result of the suggested process and compares it with different state-of-the-art processes. Finally, Section 5 gives the summary of the research, summarizing the findings and providing an idea of future studies and improvement.

## 2 Literature review

There are a number of studies that would help in developing the field of plant disease recognition, through DL paradigm.

Hemalatha and Jayachandran (2024) proposed the PDLC-ViT model, in which localization task and classification task are combined into the Multi-Task Learning (MTL) model. This training strategy was taken on top of the Plant Village dataset which introduces co-scale, co-attention, and cross-attention layers into the Vision Transformer design. Although the model has recorded impressive performance indicators, e.g. 99.97 percent accuracy and 99.18 percent Mean Average Precision, this high generalizability might be limited by the fact that the model has been trained using a single dataset, which may exclude other agricultural ecosystems and the types of disease instances.

The method proposed by Yang et al. (2024) is the LDI-NET-a network that aims at recognizing the plant type with a disease at the same time and with the level of that disease. The model has been working on the dataset of AI Challenger 2018 and has a three-phase mechanism, which is a feature tokenization process using CNNs and transformers, a token encoder to learn relationships relating to diseases severity, and a multi-label decoder fused classifier. The model showed high sensitivity in diagnosis of plant type and diseases but suffered on severity ranking, probably since it was complex to differentiate the symptoms.

AgirLeafNet is a hybrid model proposed by Saleem et al. (2024) via the use of NASNetMobile as the feature extraction model and Few-Shot Learning as the classification model. In as much as the model had an outstanding accuracy in identifying diseases in potato leaves, tomato, and mango leaves, the possibility of overfitting is a point of concern, especially when having limited-labeled data.

Shafik et al. (2024) is the representation of two models, namely PDDNet-AE, which leverages the use of an ensemble methodology in the classification of plant diseases, and PDDNet-LVE, which utilizes the use of an ensemble approach in the classification of one of the most prevalent plant disease categories: plant chemical diseases. The work by making use of PlantVillage dataset demonstrated a large degree of accuracy of 96.74 percent and 97.79 percent. The combination of several pre-trained CNN, however, can complicate the computational complexity and even prevent real-time use.

De Silva and Brown (2023) concentrated on the use of CNNs and Vision Transformers with a multi-spectral imaging-based procedure. Although the research had high levels of performance compared to its competitors, it is not high as the specified in the study and thus additional research attempting to tune the state-of-the-art approach in regards to disease detection research is required to further raise accuracy levels.

DLMC-Net is a lightweight CNN process in multi-crop plant disease recognition that is created by Sharma et al. (2023). The analysis of this model on citrus, cucumber, grape, and tomato data showed different accuracies which pointed to the possibility of performance loss when this model is applied to complex plant species and different disease expression patterns.

Li et al. (2023) developed a vision transformer called PMVT, adapted to mobile devices, reaching significant accuracy when used on wheat, coffee and rice datasets. However, the performance of the model is variable in relation to various crops, a factor that implies that there are growth areas where improvement of the model can be carried out in relation to some disease detection instances.

Lye and Ng (2023) suggested an enriched vision transformer to improve plant disease analysis performance by incorporating features and increasing stability. The proposed innovation in this method is that it generalizes well on leaves that are not in central locations in imagery, as well as that it is consistent across changing leaves orientations.

To address the shortcomings of single-teacher KD models, Amirkhani et al. (2021) presented a strong semantic segmentation framework with a Multi-Teacher Knowledge Distillation (KD) to accompany a strong semantic segmentation framework. On several datasets, they trained five different networks of lightweight CNN and used different augmentation approaches to boost the robustness and accuracy of the student model. The proposed process allowed the student model to effectively incorporate information acquired in different sources in order to increase the performance in clean and noisy conditions. Their experiments indicated that multi-teacher KD method improved between 9subfigureerr percent and 32.18 percent compared to single-teacher arrangements. However, the approach still faces limitations in terms of scalability and increased computational complexity during the training phase due to the involvement of multiple teachers. **Table 1** presents the summary of the existing methods handled by various authors.

**Table 1:** Summary of related works.

S. No	Author & Year	Technology	Results obtained	Advantages	Limitation
1	Hemalatha and Jayachandran (2024)	PDLC-ViT model with Multi-Task Learning framework and Vision Transformer	Accuracy: 99.97%, Mean Average Precision: 99.18%, Mean Average Recall: 99.11%	Enhanced localization and classification	Reliance on single dataset
2	Yang et al. (2024)	LDI-NET with CNNs and transformers	Plant accuracy: 99.42%, Severity accuracy: 88.55%, Plant-disease-severity accuracy: 87.40%	Effective feature fusion	Lower accuracy in severity detection
3	Saleem et al. (2024)	AgirLeafNet - hybrid model with NASNetMobile and Few-Shot Learning	Potato: 100%, Tomato: 92%, Mango: 99.8%	High accuracy across different crops	Potential Overfitting issues
4	Shafik et al. (2024)	PDDNet-AE and PDDNet-LVE with nine pre-trained CNNs	PDDNet-AE: 96.74%, PDDNet-LVE: 97.79%	Strong ensemble performance	High computational complexity
5	De Silva and Brown (2023)	CNNs and Vision Transformers with multispectral imaging	Accuracy: 83.3%, Precision: 90.1%, Recall: 90.75%, F1 score: 89.5%	Innovative use of multispectral data	Lower accuracy compared to contemporary methods
6	Sharma et al. (2023)	DLMC-Net with collective blocks and passage layers	Citrus: 93.56%, Cucumber: 92.34%, Grapes: 99.50%, Tomato: 96.56%	Efficient parameter usage	Inconsistent performance across different crops
7	Li et al. (2023)	PMVT (plant-based MobileViT)	Wheat: 93.6%, Coffee: 85.4%, Rice: 93.1%	Resource-efficient, Suitable for mobile devices	Variable accuracy across different crops
8	Lye and Ng (2023)	Vision transformer with enhanced features	Test accuracy: 89.58%	Robust and stable performance	Moderate accuracy compared to other methods
9	(Amirkhani et al. (2021)	Multi-Teacher KD for Robust Semantic Segmentation	Achieved performance improvement of 9%–32.18% over single-teacher models.	Improved performance in noisy and clean environments	Scalability and training complexity due to multiple teachers
	Proposed Manuscript	Heterogeneous ensemble with U-Net, Swin Transformer V2, CoAtNet, Enhanced CoAtNet, and LFHBA-based fusion	Accuracy: 99.32%, Precision/Recall/F1: 99.31–99.32%	Robust segmentation + classification, superior performance over single/ensemble baselines	Limited compliance with data protection regulations (e.g., GDPR)

## 3 Proposed methodology

**Figure 1:**
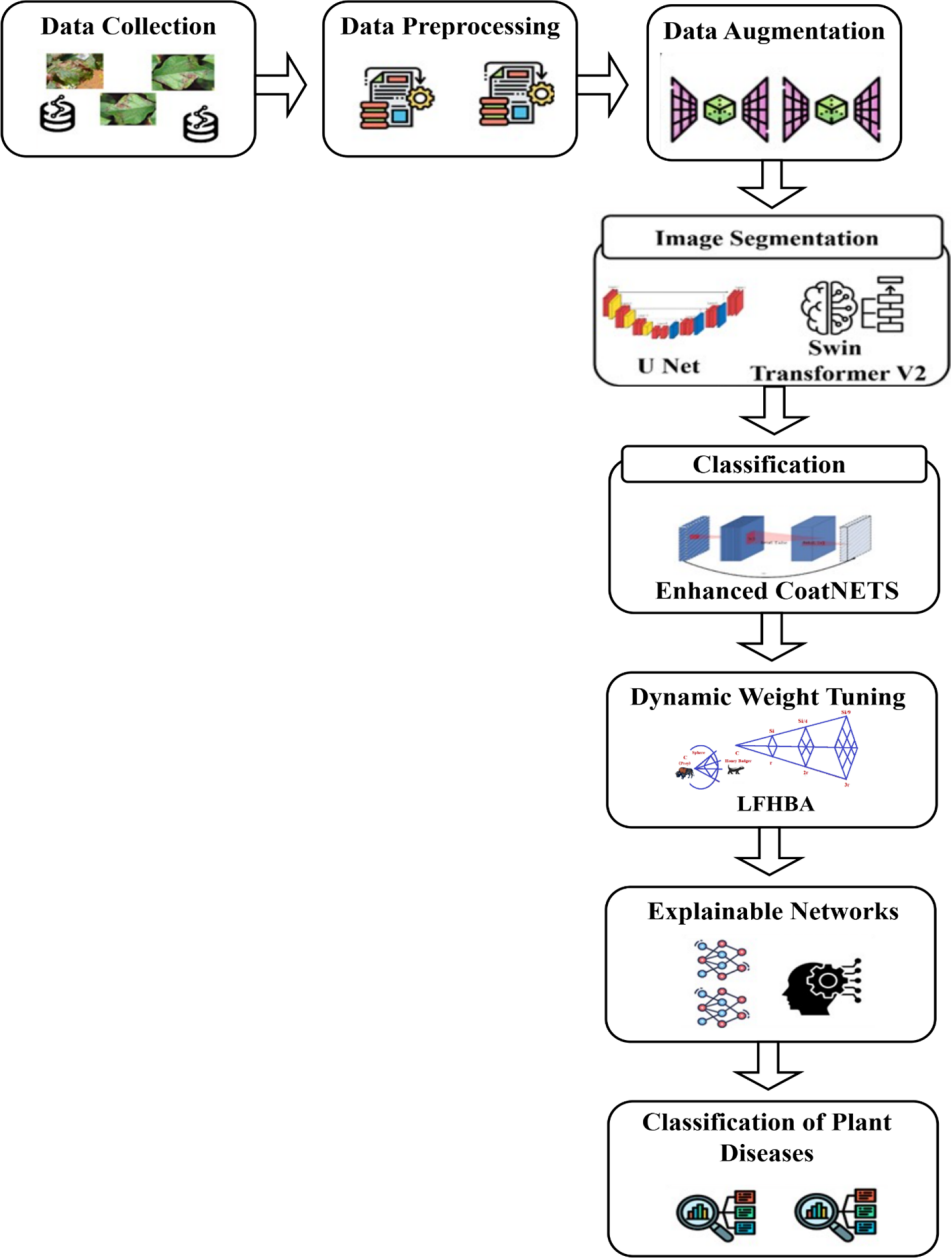
Entire architecture for the proposed approach.

**Figure 1** illustrates the overall architecture of the proposed model. To augment the data and increase the generalizability of the algorithm, various image augmentation methods are used. These consist of rotational transformations to deal with variation in leaf orientation, both horizontal and vertical flipping to reproduce natural variation in the growth of plants, scaling to reproduce variation in leaf size, and contrast adjustment to deal with variation in the lighting conditions. These additions are successful in broadening the data used to enhance the capacity of the model to identify disease patterns in diverse circumstances.

A hybrid model of U-Net and Swin Transformer V2 is used to segment disease-affected regions. U-Net offers high-spatial resolution to support the accurate local segmentation, and Swin Transformer V2 offers the global contextual information that can be used to effectively outline the diseased regions. The segmentation stage splits out areas of interest in a background, which is ready to extract features and classify them.

CoAtNet and Enhanced CoAtNet are used to extract features and classify them, by incorporating the convolutional feature extraction with transformer-based global context learning. This hybrid design captures the intricate hierarchical trends in the segmental areas and enhances the correctness of the classifications of diseases.

In order to increase strength and minimize possible biases, a heterogeneous ensemble decision fusion plan is utilized. The ensemble combines the forecasts of several transformer-based models, where the Levy Flight Honey Badger Algorithm (LFHBA) dynamically adapts the weights used in the fusion process in order to maximize precision in the classification and the general functionality of the ensemble.

GRAD-CAM visualizations are used to provide model explainability, which is used to show regions of input images that have the greatest effect on the model predictions. This makes it more transparent and enables the user to know how the decisions to classify were made.

The suggested framework is assessed with the help of conventional metrics such as accuracy, precision, recall, and F1-score. These evaluations confirm the reliability and effectiveness of the approach, demonstrating its readiness for practical agricultural deployment. Ultimately, the system can be implemented in real-world conditions, providing farmers with a convenient tool for on-the-fly diagnosis and management of plant diseases, translating theoretical gains into tangible benefits for the agricultural community.

### 3.1 Materials and methods

**Figure 2:**
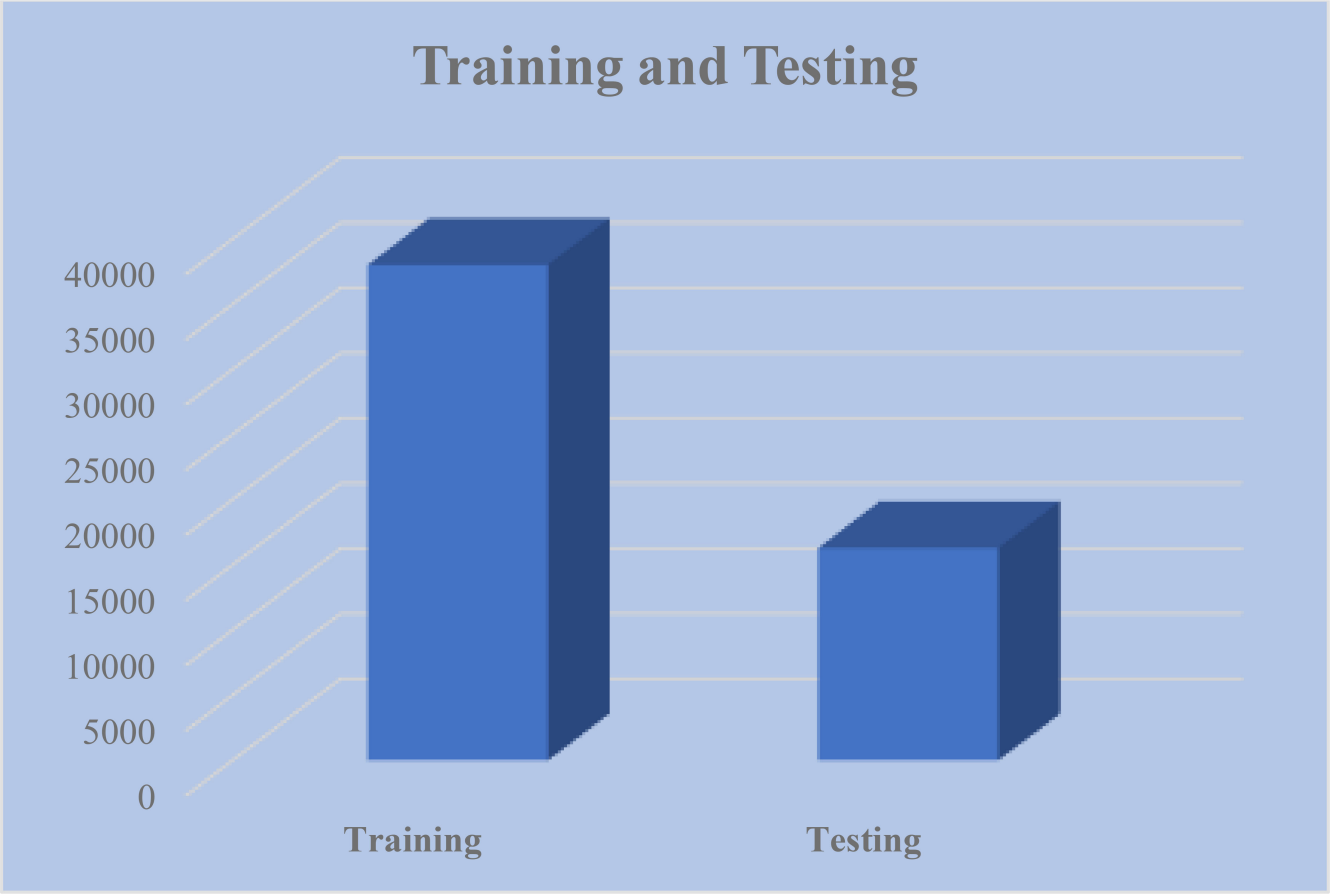
Datasets images utilized for training and testing the proposed approach.

**Figure 3:**
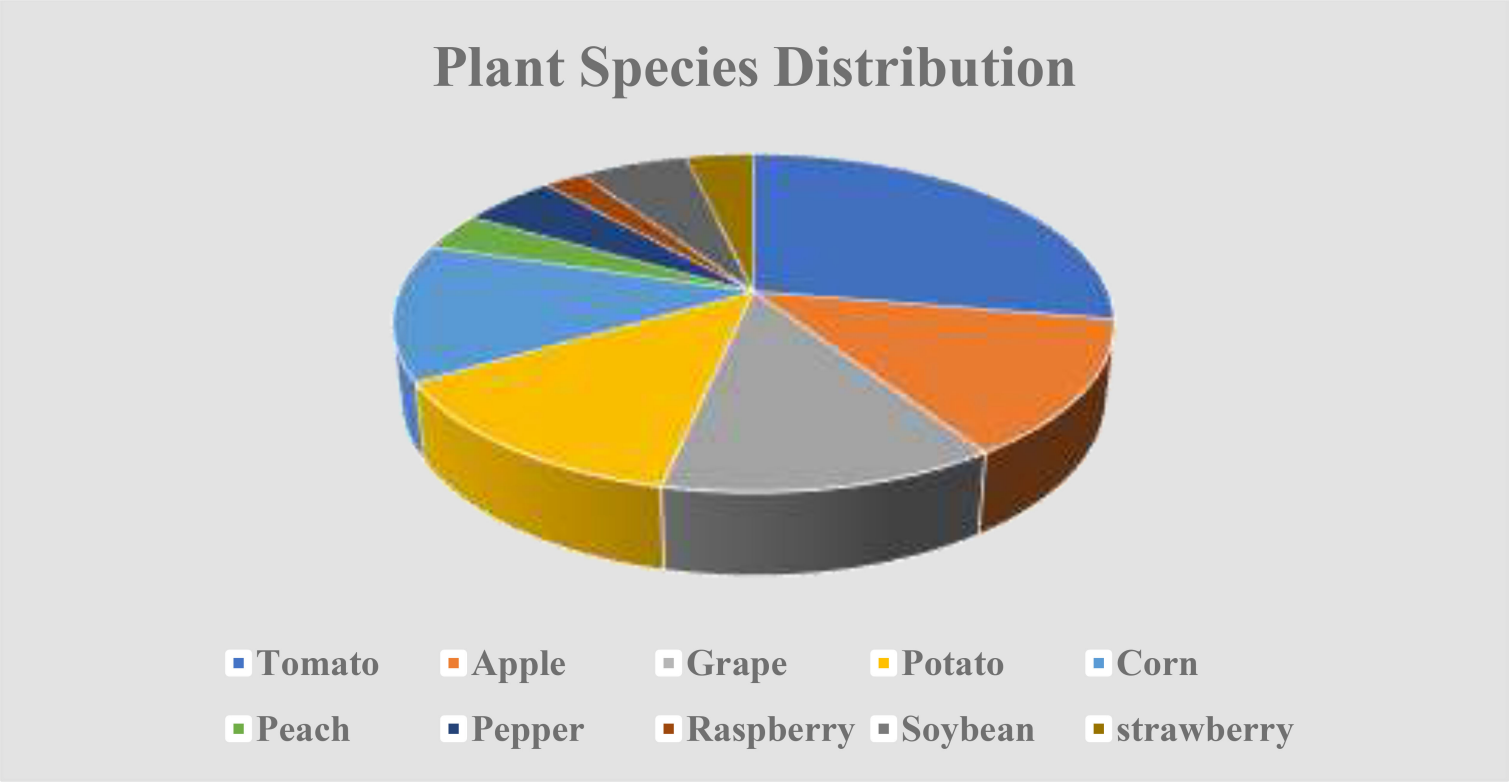
Different plant species distribution.

The reason why this study is conducted on the Plant Village Dataset is that it is a well-known resource that could be used in the field of plant disease classification. The data consists of 54,305 images as can be seen in **Figure 2**, in 38 different classes representing different plants and their condition (healthy or diseased). The data set consists of 17 different plant species of varied disease pattern as shown in **Figure 3** like Apple with Apple Scab, and Apple Cedar Rust diseases, and Tomatoes with Tomato Late blight and Tomato Mosaic Virus. Presentation of images over these classes permits an extensive analysis and training of the presented model.

### 3.2 Image preprocessing

**Figure 4:**
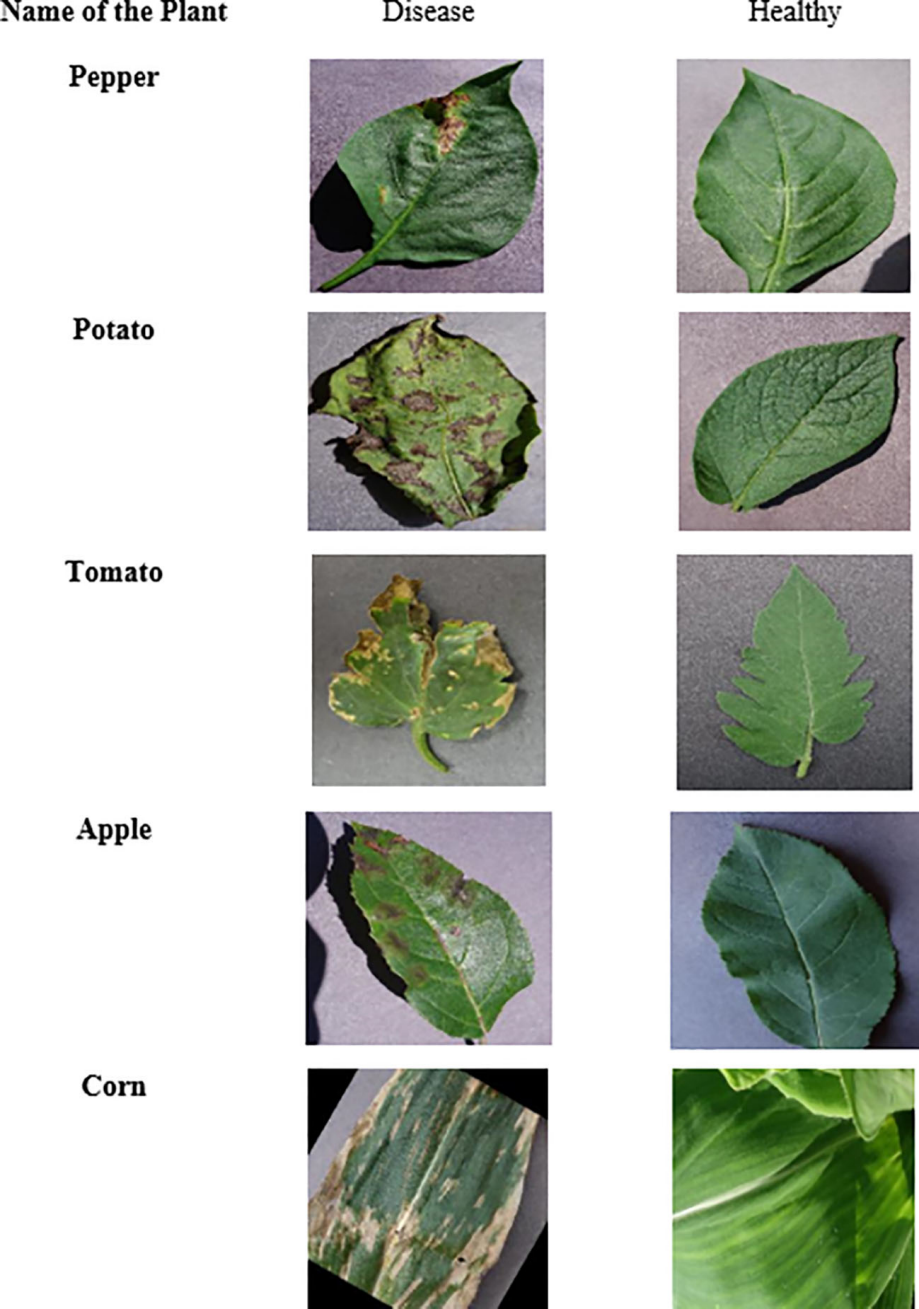
Dataset details utilized for the recommended approach.

To ensure the model’s robustness to variations in input data, a preprocessing pipeline is established. This includes resizing images to a consistent 224 × 224 pixels, as shown in **Figure 4**, and applying a combination of normalization and augmentation procedures, like grayscale conversion, rotation, flipping, and brightness adjustment. These steps are essential in preparing the dataset for the subsequent stages of segmentation and classification.

### 3.3 Image augmentation

**Figure 5:**
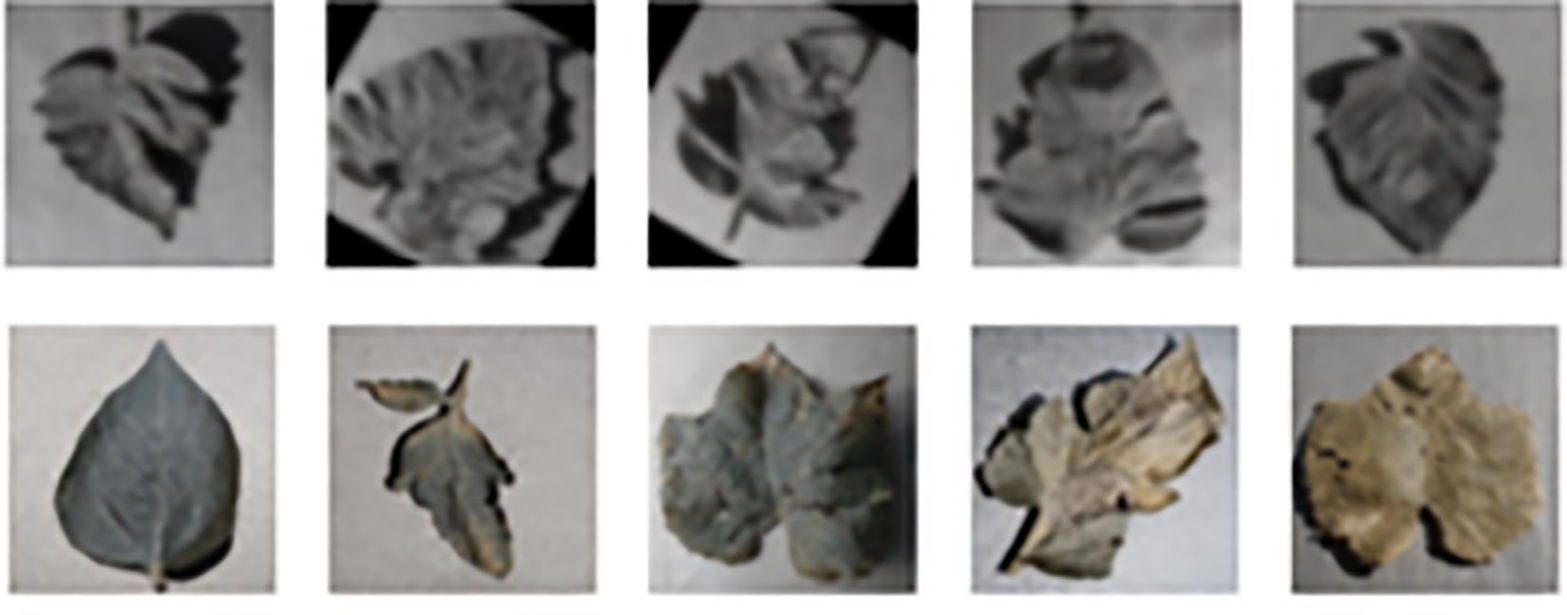
Processed and augmented images.

The augmented and processed images are presented in **Figure 5**, demonstrating the diversity and complexity of the data that the approach must handle. This comprehensive approach to data preparation and model design underscores the research’s commitment to developing an effective and reliable tool for plant disease diagnosis.

### 3.4 Image segmentation methodology

**Figure 6:**
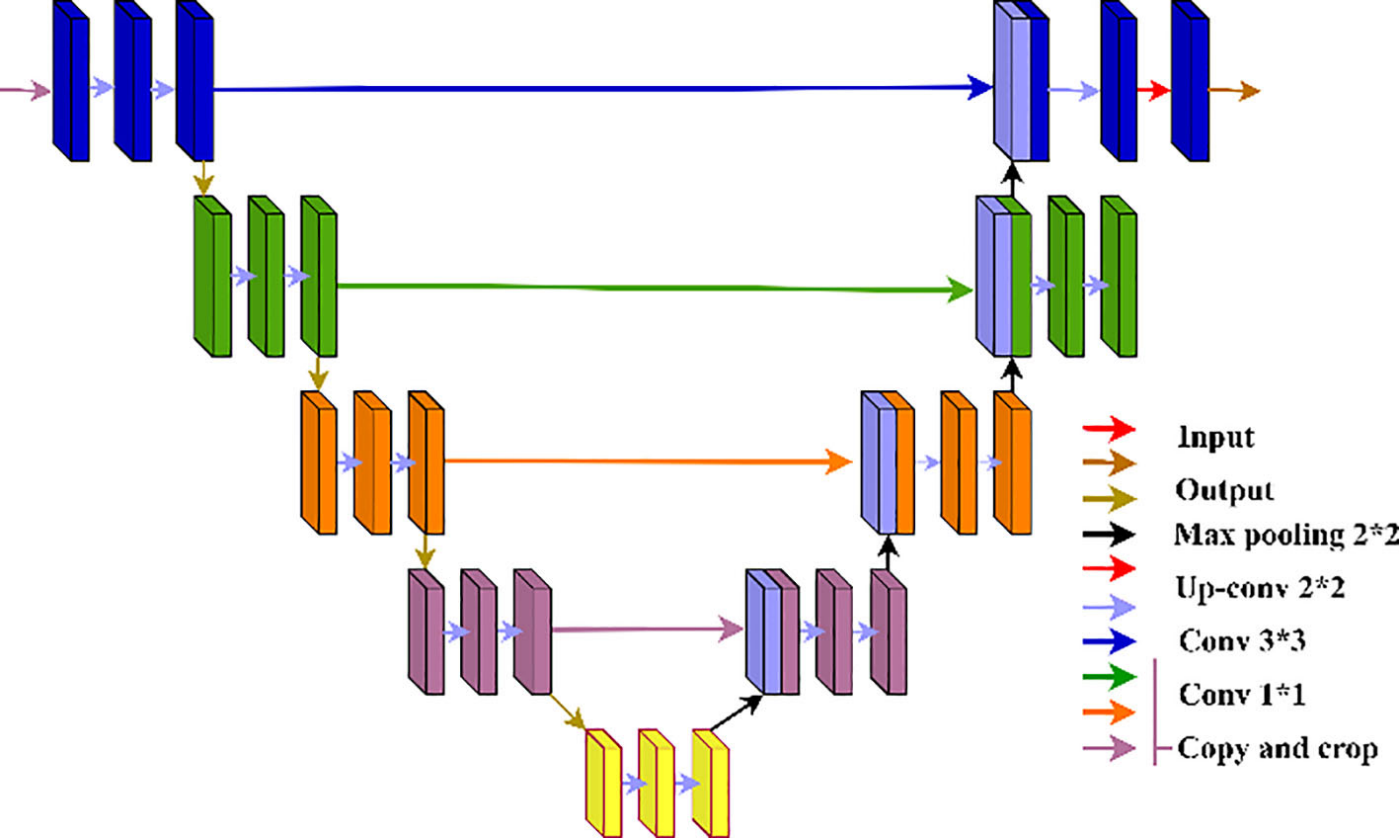
Structure for the U-Nets.

The segmentation process plays an important role in the isolation of the disease affected areas within the images. In the study, a U-Net based architecture is used, which is effectively applied in biomedical image processing as shown in **Figure 6**. This architecture has an encoder-decoder design that has skip interconnections to enable it to retain high-resolution attributes and extract deep features. The encoder or contracting path constitute convolutional layers then with ReLU activations and max-pooling to eliminate the spatial dimensions of the input image successively.

#### 3.4.1 U-Net framework

The U-Net is a leading DL methodology that can be employed to achieve image segmentation tasks especially when it comes to biomedical image analysis. It is an encoder-decoder design with convolutional neural networks (CNNs) and is characterized by the inclusion of skip connections that aid in maintaining the spatial resolution as well as at the same time perform advanced feature extraction. **Figure 6** presents a graphic illustration of this framework.

The contraction path of the U-Net is a sequence of convolutional blocks, followed by a rectified linear unit (ReLU) activation function, and a max-pooling operation as presented in Equation 1. Max-pooling operation (MaxPool) is used to reduce the spatial dimensions of the feature maps as explained by Equation 2.

(1) $$F_{l}=ReLU(W_{l}*F_{l-1}+b_{l})$$

(2) $$F_{pool}=MaxPool(F_{l})$$

The bottleneck layer is placed at the bottom position of the network and it is defined by the large count of features map even though it has restricted spatial dimensions. Before this information is sent to the decoder, the abstract and high level features learn through this layer.

The decoder, or the expansive path, employs transposed convolutions to restore the original image resolution, as illustrated in Equation 3. The transposed convolution, often termed deconvolution, enlarges the spatial dimensions of the feature maps ( $F_{l}$).

(3) $$F_{l}=ConvTranspose(F_{l-1},\;W_{l})$$

A notable feature of U-Net is the utilization of skip connections (F_skip_​), which concatenate the feature maps from the encoder with their corresponding maps in the decoder, as detailed in Equation 4. This approach equips the decoder with the ability to exploit high-level abstract features alongside low-level spatial information.

(4) $$F_{skip}=concat(F_{encoder},F_{decoder})$$

#### 3.4.2 Swin Transformer

**Figure 7:**
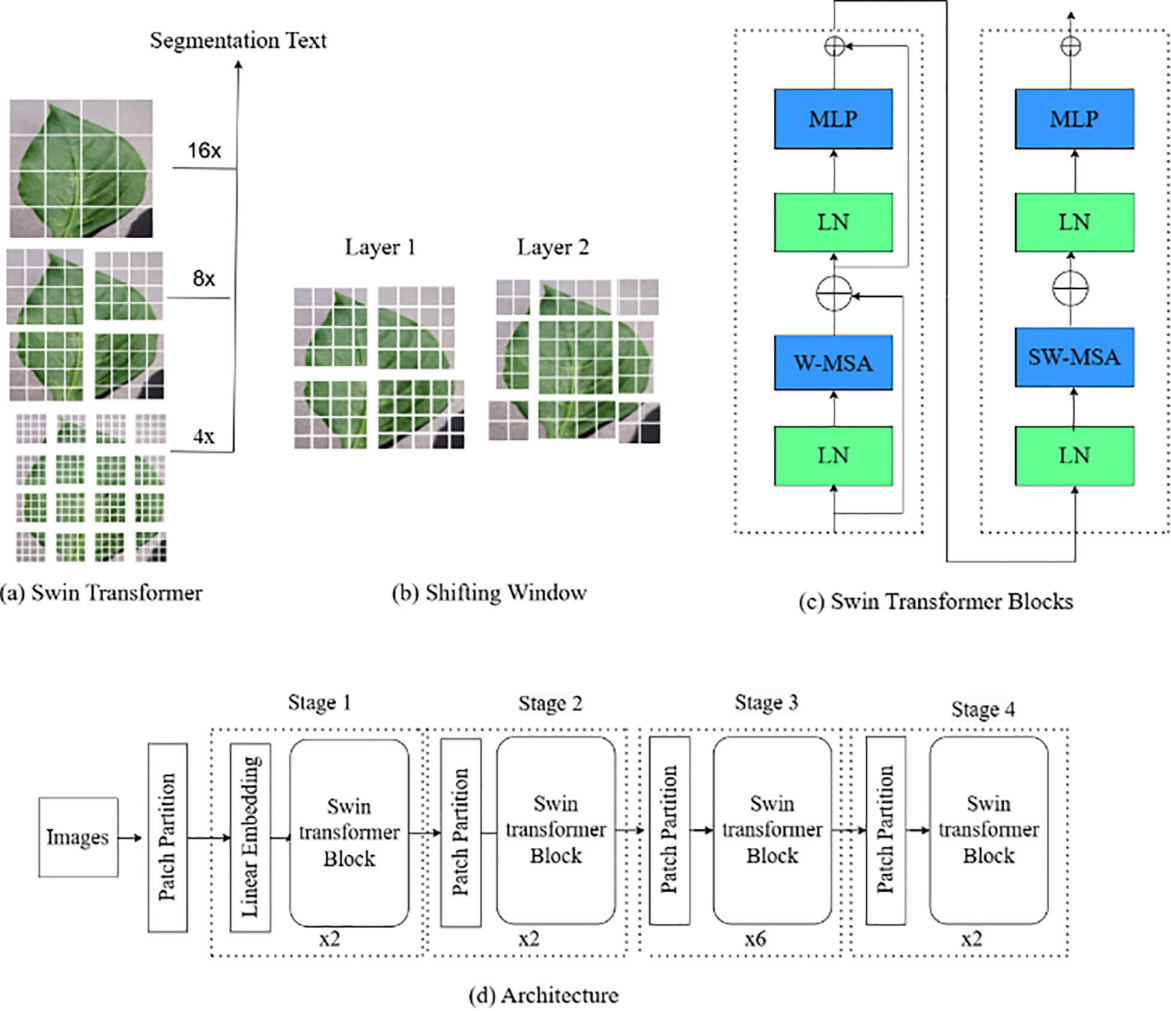
Network structure for SWIN-V2.

The Swin Transformer (ST) V2 is an advanced version of TNN model designed for image classification and segmentation tasks. Unlike traditional CNN-based architectures, Swin Transformer V2 leverages a hierarchical structure of local windows and shifts to better capture both local and global context in the image, as presented in **Figure 7**. The model improves upon the original Swin Transformer by enhancing the efficiency, scalability, and performance on large-scale vision tasks.

The initial step involves patch partitioning ( $Z_{0})$ and linear embedding, where the input image I is divided into patches of size H×W, as stated in Equation 5. These patches are then projected into a D-dimensional linear space via a linear transformation layer.

(5) $$Z_{0}=Linear(Flatten(I))$$

The hierarchical transformer blocks are the core of the model, integrating Window-based Multi-Head Self-Attention (W-MSA) and Shifted Window Multi-Head Self-Attention (SW-MSA) to efficiently capture local and global information. The multi-head self-attention mechanism is outlined in Equation 6, where Q, K, and V denote query, key, and value matrices.

(6) $$Attention(Q,K,V)=softmax(\frac{QK^{T}}{\sqrt{d_{k}}})$$

W-MSA, detailed in Equation 7, operates on non-overlapping windows, thereby enhancing computational efficiency by limiting the attention of each token to its windowed counterparts.

(7) $$Z_{l}^{W-MSA}=W-MSA(X_{l})$$

SW-MSA, introduced to capture cross-window interactions, is presented in Equation 8. This mechanism involves shifting the window $\left(Shift(X_{l-1})\right)$ positions between layers to facilitate attention across distant patches, thereby enhancing the model’s capacity to understand global context.

(8) $$Z_{l}^{SW-MSA}=SW-MSA(Shift(X_{l-1}))$$

Each block within the Swin Transformer V2 also includes a multi-layer perceptron (MLP) for nonlinear transformations and Layer Normalization (LN) to stabilize the training process, as expressed in Equations 9, (10). $Z_{l}^{Att}$ is the output of this weights $W_{1}$ & $W_{2}$ matrices.

(9) $$Z_{l}^{MLP\;}=ReLU(W_{1}Z_{l}^{Att}+b_{1})W_{2}+b_{2}$$

(10) $$Z_{l}^{Norm}=LN(Z_{l}^{MLP\;})$$

Finally, a kind of down sampling that patch merging is then performed after the transformer block to create the hierarchy feature map. This is one operation, expressed by Equation 11 which convolves neighbouring patches so as to reduce spatial dimensions and expand the number of channels.

(11) $$X_{l+1}=\;Merging(X_{l})$$

The final output, a global feature representation, is processed through a classification head for image classification tasks.

### 3.5 Classification and feature integration

#### 3.5.1 CoAtNet: convolution and attention hybrid network

**Figure 8:**
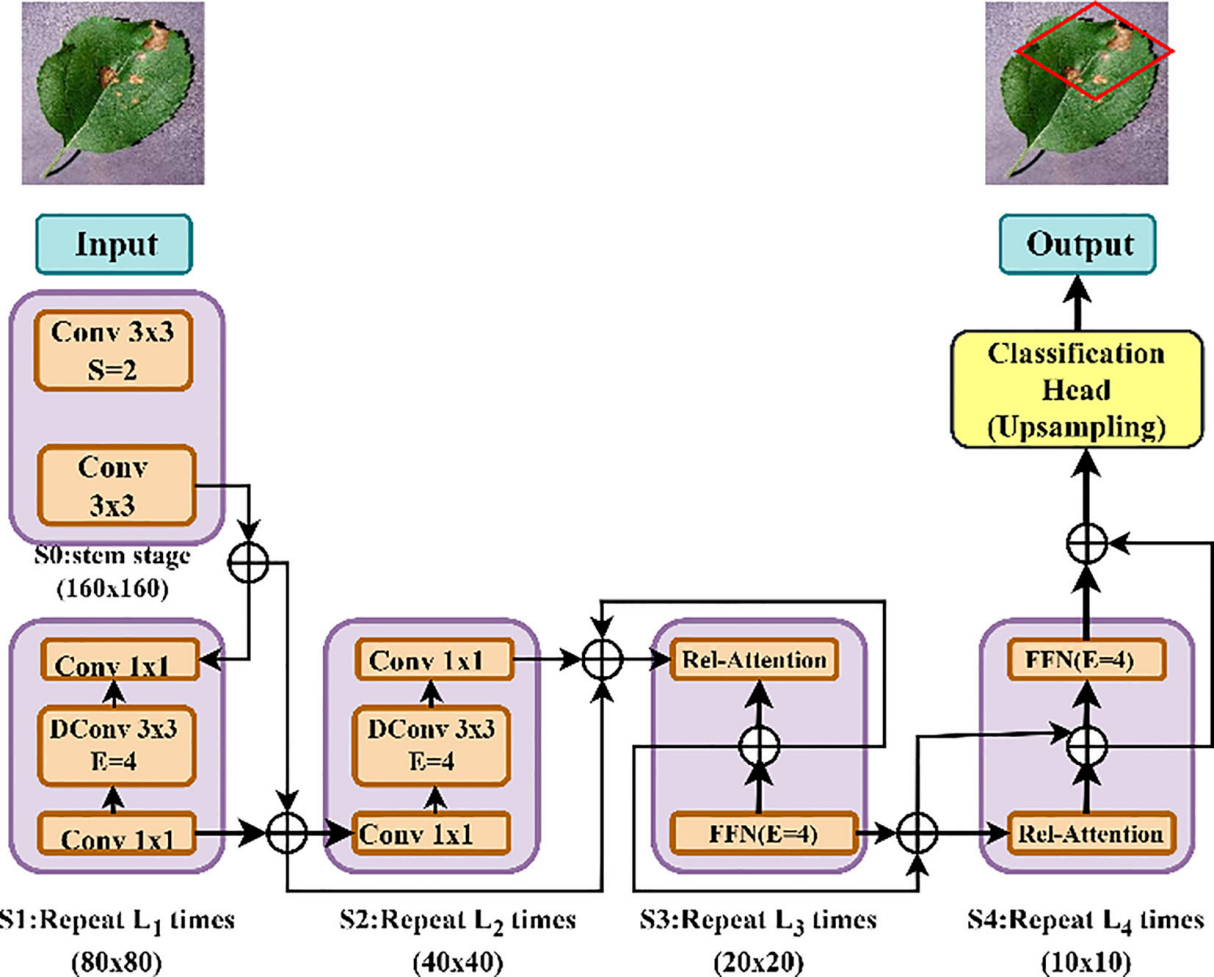
CoAtNet model architecture combining convolution and attention mechanisms.

CoAtNet is a new DL architecture that can effectively integrate the goodness of Convolutional Neural Networks (CNNs) and those of Transformer-based attention. Such a hybrid system is in a hierarchical manner, where the lower levels of the structure deal with CNN-based local feature extraction, and the higher rank of the system would be to follow self-attention to capture global dependencies, as indicated in **Figure 8**.

##### 3.5.1.1 Convolutional feature extraction

Procedures of convolutions are adopted in the layers of CoAtNet early steps to pull out and identify later unknown features in form of edges, textures and color changes detected in plant leaves portrayed in Equation 12. This process will ensure that important spatial information has been properly recorded prior to shift to transformer-based layers.

(12) Y= σ(W*X+b)

where X is the input image, W represents the convolutional kernel, b is a parameter of a bias, convolutional process is represented by an operator *, and the activation function is denoted by sigma and can include ReLU or GELU (Gaussian Error Linear Unit).

##### 3.5.1.2 Self-attention mechanism

The unit of self-attention plays a critical role in determining and assigning weights to relevant points which are important in differentiating various classes. The similarities of the locations of features are compared and used to compute the attention weights; this is done as illustrated in Equation 13. Through this process, the network learns contextual relations as well as feature representation improvement.

(13) $$Z=Softmax(\frac{QK^{T}}{\sqrt{d_{k}}})V$$

where Q, K and V represents query, key and value matrices, which are extracted out of the feature maps; d k represents the dimension of the key matrix. To bring the attention scores in the range of [0, 1], the Softmax function is used to force the most relevant aspects.

As a result of the addition of self-attention mechanisms, CoAtNet improves its ability to detect complex patterns of diseases possible tissue distribution on various parts of the leaf surface at many times. This increased feature representation will help in the better understanding of the contributor in the field of plant pathology thus helping in the development of precision agriculture and plant health diagnostic systems.

##### 3.5.1.3 Feedforward layer within transformer blocks

After going through the self-attention phase of Transformer blocks, a FeedForward neural network (FFN) is superseded to further construct the feature representations that have been derived to be more accurate. As mentioned in Equations 13–21, This step involves two linear transformations with an activation function in between which is non-linear:

(14) $$F(x)=\;\sigma (W_{2}(ReLU(W_{1}X+b_{1}))+b_{2})$$

In this case $W_{1}$ and, $W_{2}$ represents weight matrices, and $b_{1}$ and $b_{2}$ represents biases and, the activation estimate is denoted by s. The operation is used to boost the discrimination ability of the model between healthy and diseased leaves of a plant.

This element exercises a significant role towards improving the discriminative ability of the model under study of differentiating the healthy and the pathologic plant foliage. A combination of the CNN skill of local feature map extraction with the Transformer capacity to learn global representations results in the excellence of CoAtNet in a classification setting. This is attained at the same time retaining computational efficiency which is a serious aspect in terms of practical applicability and scalability.

#### 3.5.2 Enhanced CoAtNet: multiscale feature aggregation for robust disease identification

**Figure 9:**
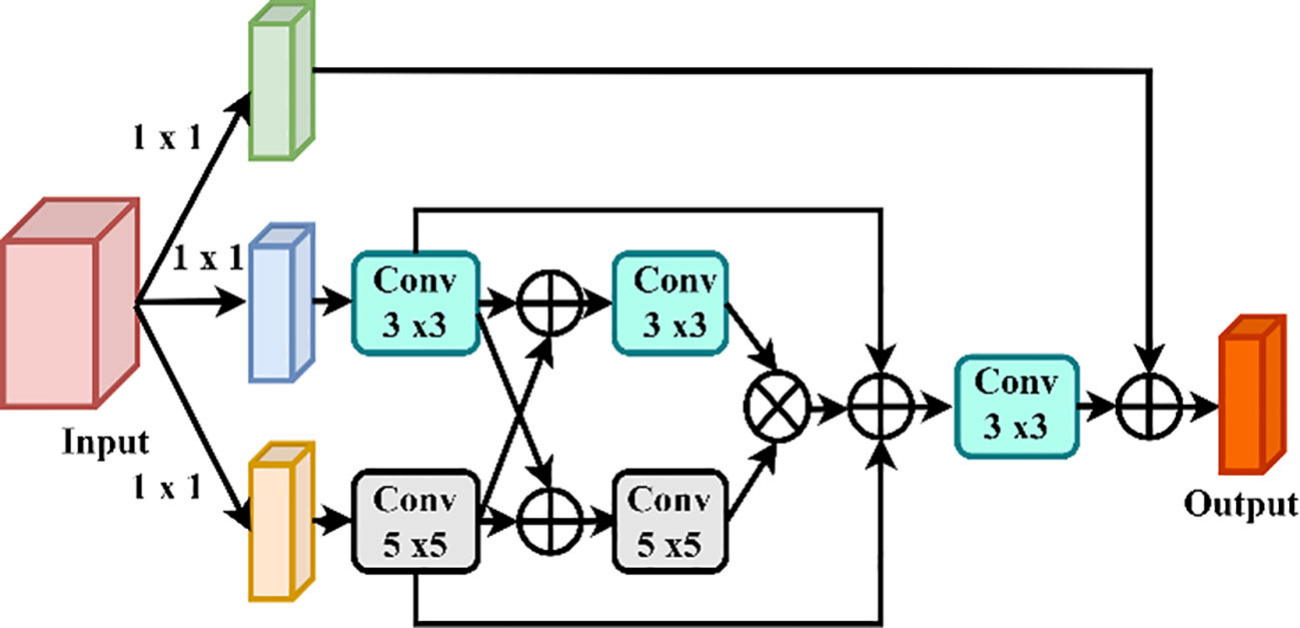
Multiscale feature aggregation module architecture.

To solve the multidimensionality of the plant disease manifestation, the Enhanced CoAtNet presents a multiscale feature aggregation, and an adaptive attention module, based on improvements to the original CoAtNet model. **Figure 9** clarifies the details of the Multiscale Feature Aggregation Module, offering a pictorial explanation of the complex mechanism through which the features are synthesized and refined on multiple spatial and temporal scale, hence introducing a deeper and more detailed way to process the procedure of representation learning.

##### 3.5.2.1 Multiscale feature fusion

The fact, that the plant leaves have lesions of different sizes and severity, requires a model that provides the integration of multiple scale features. Enhanced CoAtNet uses multiscale feature aggregation approach, which is characterized by:

(15) $$F_{multi}=\sum_{i=1}^{N}\alpha_{i}F_{i}\;$$

Where $F_{i}$ denotes feature maps at differing scales and $\alpha_{i}$ are learnable scaling coefficients which determines the contribution of a given scale. N represents the amount of feature scales to consider. This process enhances the ability of the approach in acquiring the small scale patterns of lesion and large scale of the disease spread and this provides enrichment in accuracy in classification and segmentation of lessons.

##### 3.5.2.2 Adaptive attention module

Enhanced CoAtNet aims at maximizing feature extraction by enabling an adaptive attention mechanism to dynamically balance convolutions and self-attention operations:

(16) $$A(x)=\;\lambda_{1}\;.\;Conv(x)+\;\lambda_{2}\;.\;Self-Attention(x)$$

In this case, learnable weight factors are $\lambda_{1}$ and $\lambda_{2}$: they correct the prominence of convolution-based feature extraction (Conv(x)) and self-attention (Self-Attention(x)). This allows the approach to flexibly attend to relevant features, which make it more robust against the adverse effects like occlusions, light changes and background noise.

##### 3.5.2.3 Regularized feature representation to mitigate overfitting

Enhanced CoAtNet encapsulates dropout within transformer blocks to avoid the problem of overfitting that is common when dealing with large datasets such as Plant Village:

(17) $$\hat{Z}=Dropout(Z)$$

Where Z of attention-weighted features representation. Also, it implements batch normalization:

(18) $$F_{norm}=\;\gamma (\frac{F-\;\mu }{\sigma })+\;\beta$$

In this case, both μ and σ will represent the mean and the standard deviation to the feature activations whereas γ and β will be learnable parameters of the scale and shift. These regularization forms improve the generalizability of the model and therefore, the model can be used to identify plant diseases in a varying environmental setting.

Our architecture two stage ensemble using CoAtNet and Enhanced CoAtNet is a key to the overall high accuracy and robustness of diagnosing plant diseases. Our model presents a retrieving strategy to deal with plant disease analysis due to using complementary power of CNNs to extract local features and Transformers to exploit long-range correlations. The multiscale aggregation property of Enhanced CoAtNet is necessary to identify the diseases at multiple levels and the adaptive attention mechanism updates the concentration towards the significant features. Moreover, dropout regularization and batch normalization are used, which counter-acts the overfitting you said, and the performance on new data is better.

This approach uses U-Net and Swin Transformer V2 to segment the image and then uses an enhanced CoAtNet as its classifier, achieving remarkable results compared to the current deep learning-based algorithms when tested on the PlantVillage dataset. This kind of synergistic integration is able to isolate diseased areas before classification which makes the proposed system very precise and reliable in regard to the automated detection of plants diseases.

### 3.6 Meta-heuristic decision fusion using the levy flight-based honey badger algorithm for enhanced diagnostic accuracy

To overcome the drawback of the existing decision fusion methods i.e. majority voting or probabilistic weighted averaging, we incorporate the Levy Flight-Based Honey Badger Algorithm (LFHBA) into our model. This meta-heuristic technique improves the feature selection and classifier fusion, and make our model match dynamically to different plant diseases scenarios.

The Honey Badger Algorithm (HBA) is a bio inspired optimization process that was based on the hunting and searching patterns of the honey badgers (Hashim et al., 2022). It has two broad phases which are exploration and exploitation of finding new and refinements of old solutions. The update of the position of HBA is provided by:

(19) Xt+1=Xt+r.(P−Xt) . e−tT

The $X^{t+1}$ and $X^{t}$ denote the updated and current position of the candidate solution, P is the best solution found, r is random moving factor and T is the number of iterations being done.

In order to improve HBA, we integrate the Levy Flight exploration that makes it easier to leave local optima by jumping with random long moves:

(20) $$S=\;\mu \frac{\tau (1+\;\beta )\text{sin}(\pi \beta /2)}{\tau ((1+\;\beta )/2)\beta 2^{(\beta -1)/2}}.\frac{1}{|\text{v}{|}^{1/\beta }}$$

In this case, S is the step size, 0 - a scaling parameter, v - an (N(0,1)) distributed variable. Considering the LFHBA in our ensemble, we heuristically calibrate the weights values of Neural Networks such as CoAtNet, Enhanced CoAtNet and Swin Transformer V2 according to the level of confidence of the classifier in the sequence:

(21) $$F=\sum_{i=1}^{N}w_{i}C_{i}(X)\;$$

N is the number of classifiers, $C_{i}(X)$ is the output of classifier i with input X and $w_{i}$ is the adaptive weight to be optimized by LFHBA. The adaptive weighting scheme helps the model to give importance to the best predictions and it reduces the effect of the less accurate ones. It is the capacity of LFHBA to balance exploration and exploitation that has been important in arriving at high classification accuracy with a vast number of disease classes and leaf texture, thus making the model more pragmatic in real-life situations.

The combination of LFHBA helps the overall robustness and accuracy of our decision fusion procedure, and it is better than conventional ensemble models. Such an optimization algorithm is essential in obtaining state-of-the-art performance in automatic diagnosis of plants diseases and as such our optimization algorithm is well poised to overcome the realities of such complex systems in real life.

## 4 Results and considerations

### 4.1 Implementation aspects

Our model was fully implemented using the Python 3.19 platform that incorporates the critical packages, including Matplotlib to visualize data, Pandas to manipulate data and NumPy and Seaborn to perform numerical computations and statistical visualization, respectively. The introduced experimental setup was run on the high-performance PC workstation with Intel i7, an NVIDIA Tesla 1024 GB GPU to improve the speed of extensive computation, and 16GB of RAM that works at a speed of 3.2 GHz. **Table 2** presents the training configurations and parameter settings used to develop and optimize the proposed approach.

**Table 2:** Training configuration and parameter settings for the proposed approach.

S.No	Hyperparameters	Descriptions
01	Initial Learning Rate	0.001
02	Batch Size	32
03	Optimizer	Adam
04	Number of Epochs	200
05	Activation Function	Softmax

### 4.2 Assessment criteria

The effectiveness of the proposed method was examined using a set of critical performance indicators, that is, precision, accuracy and specificity, F1-score and recall. The metrics were employed to compare them with state-of-a-art ensemble feature selection procedures in order to highlight the exceptional strengths of our approach. Moreover, a comprehensive analysis of the cost implications of the metrics was undertaken to prove its resource-efficient character. The formulas used to compute these measures exist in **Table 3**.

**Table 3:** Performance measures utilized in the evaluation.

SL.NO	Performance measures	Expression
1	Accuracy	$\frac{TP+TN}{TP+TN+FP+FN}$
2	Recall	$\frac{\text{TP}}{\text{T P}+\text{FN}}\text{x}100$
3	Specificity	$\frac{TN}{TN+FP}$
4	Precision	$\frac{TN}{TP+FP}$
5	F1-Score	$2.\frac{Precison*Recall}{Precision+Recall}$

TP & TN are True Positive & negative, FP & FN are False Positive& negative.

The confusion matrix is the prediction that is broken down into True Positive (TP), False Positive (FP), False Negative (FN) and True Negative (TN). True Positive (TP) is an occurrence in which the model properly detects and validates the existence of a positive condition and is in harmony with the real positive status of the instance concerned. In its turn, FP is defined by the false positive identification, i.e. classifying a negative case as positive. At the other end of the scale there is a FN, which occurs when the corrective diagnosis is not made because the incorrect feedback is produced, the true condition is absent. Finally, a TN represents the successful prognostication of a negative case, which is associated with the actual lack of the examined condition. These factors form a fundamental part in defining the effectiveness and accuracy of predictive models in various facets of study.

### 4.3 Experimental findings

**Figure 10:**
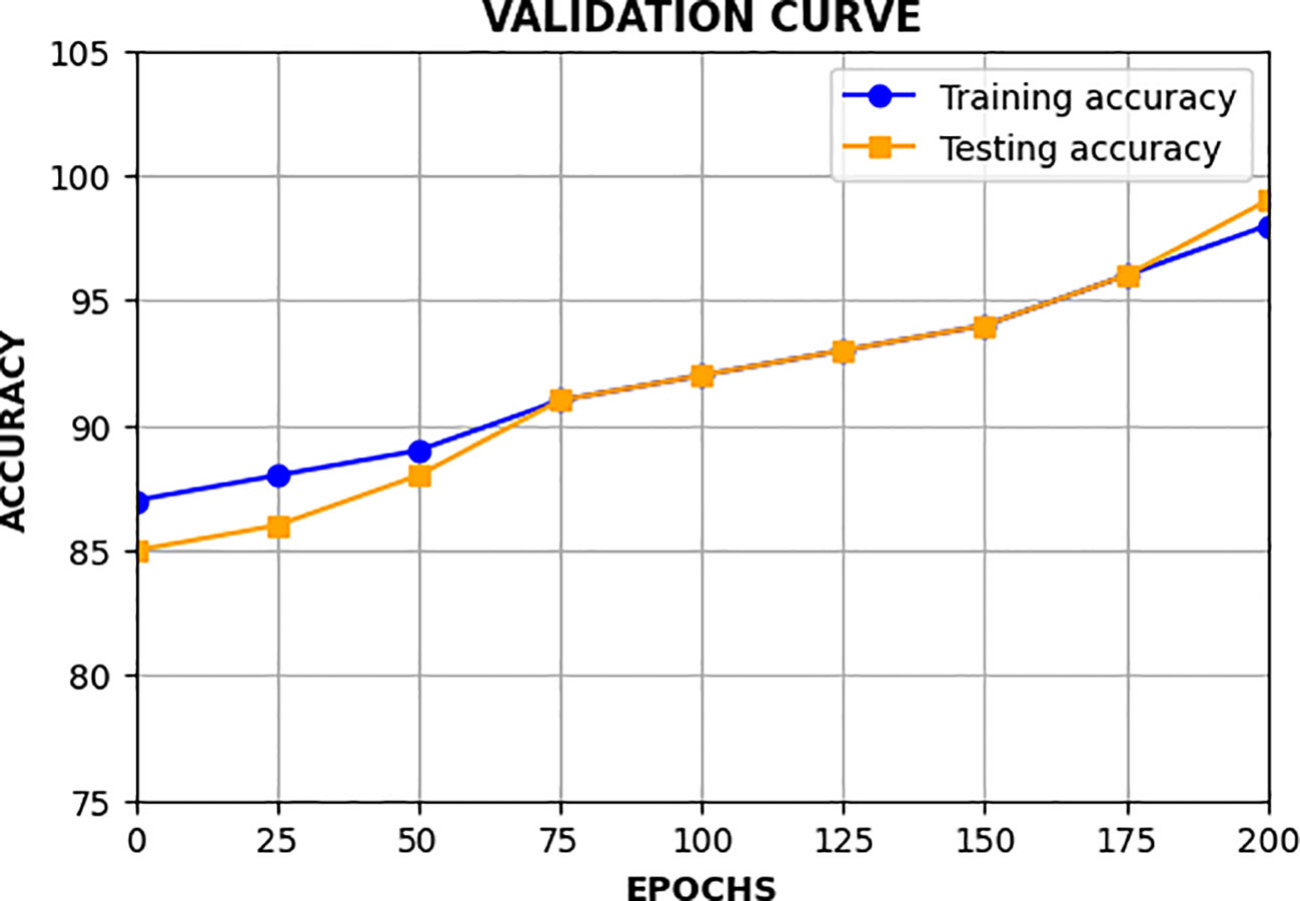
Validation performance of the proposed algorithm.

**Figure 10** demonstrates validation performance of the suggested approach, which demonstrates its ability to maintain stable performance regardless of various settings. Such a consistency also evinces the confidence and efficacy of the algorithm that will also provide consistent results regardless of any fluctuations in the operational conditions and points out its versatility concerning a wide range of applications.

### 4.4 Statistical analyses

Compared the suggested model with other existing plant disease diagnosis systems to determine the effectiveness of the proposed model; **Table 4** is given below. The outcomes confirm the effectiveness of our model regarding accuracy and efficiency, which seems especially high, considering the absence of the dependence on specialized hardware. This characteristic makes our solution an affordable and universally available feature to different user groups.

**Table 4:** Performance metrics of the different optimization approach using PlantVillage dataset.

Algorithms	Performance metrics				
	Accuracy	Precision	Recall	Specificity	F1-score
VGG16	0.802	0.778	0.791	0.762	0.747
DenseNet-77	0.817	0.819	0.822	0.843	0.811
CDCNN	0.814	0.813	0.831	0.818	0.847
Mobile ViT	0.802	0.803	0.835	0.829	0.838
CNN	0.901	0.889	0.868	0.881	0.867
Proposed Model	0.993	0.993	0.993	0.993	0.993

**Figure 11:**
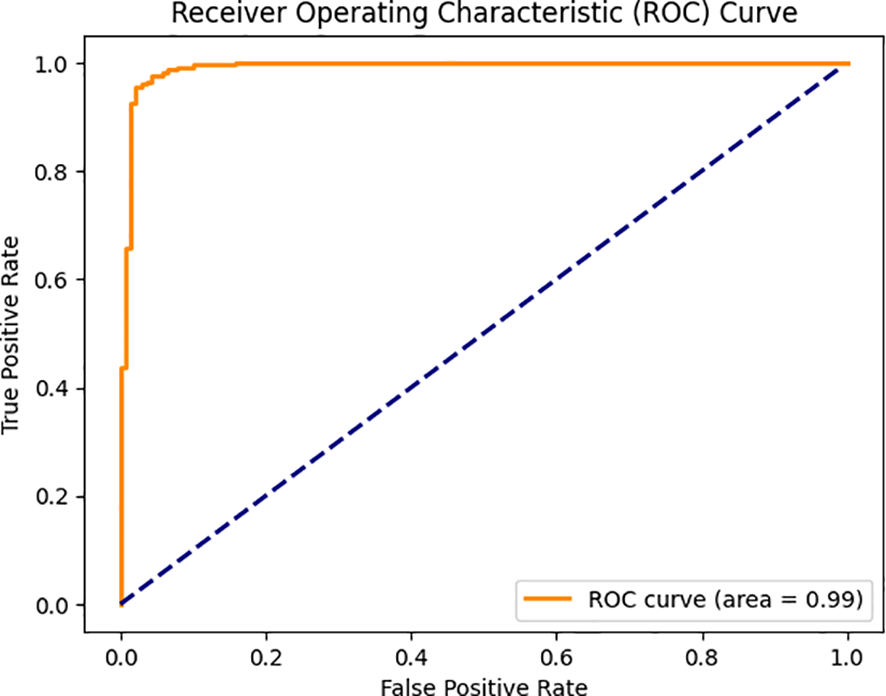
ROC curve for the proposed algorithm.

**Figure 11** displays receiver operating characteristic (ROC) curve with respect to the classification task of diagnosis of plant diseases. This is a curve that describes the behavior between True Positive rate (TPR) which describes the ratio of the correctly identified diseased samples and False Positive Rate (FPR) that describes the ratio of incorrectly labeled healthy ones, into diseased samples over a scale of classification thresholds. The visualization is applied in order to assess the discriminative ability of the approach in differentiating health and disease conditions among the botanical specimens.

**Figure 12:**
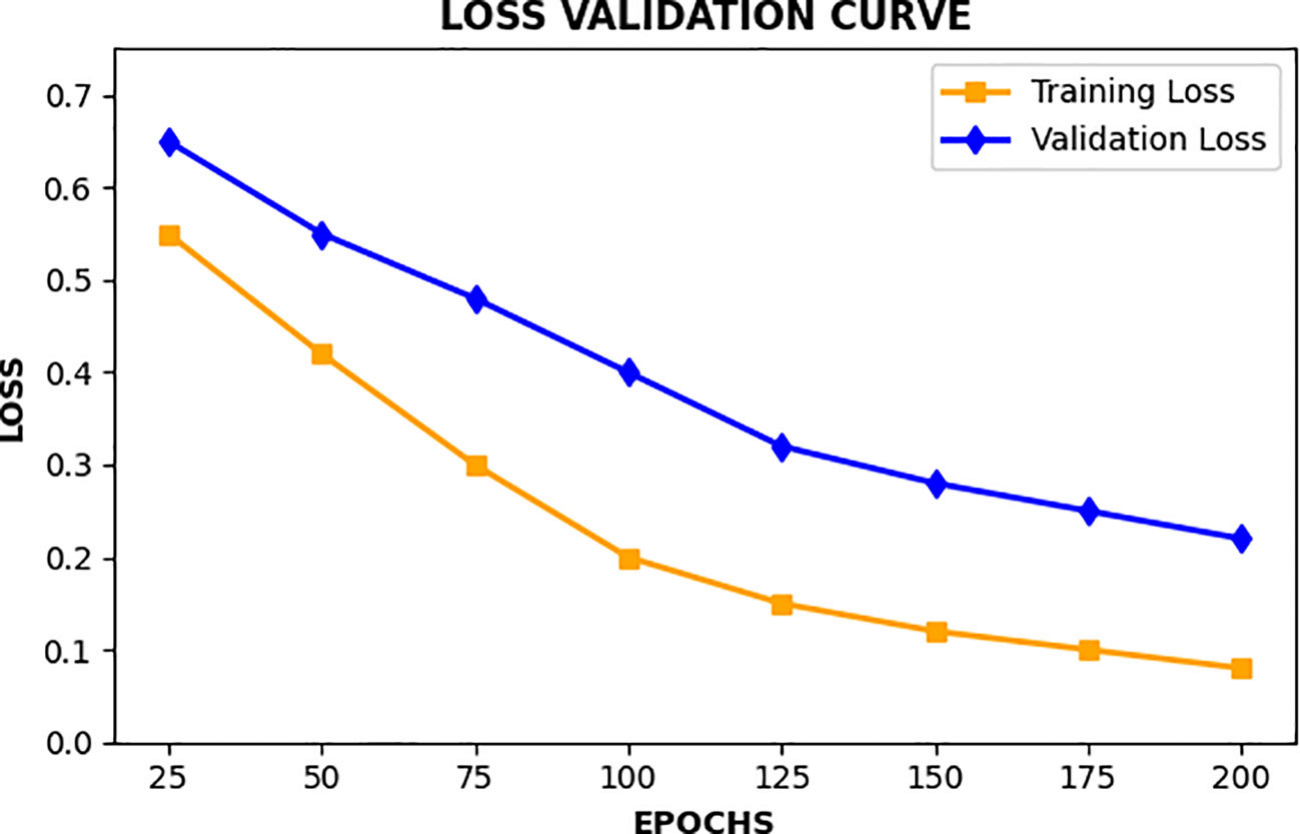
Line plots of the training and validation loss values over several training epochs.

**Figure 12** shows line plots where we can look at how the values of training loss and validation loss changes throughout many epochs. These plots provide a clue about the time dynamics of the performance of the approach. The decrease in training loss shows the process of optimization that the model is going through and the validation loss gives us the idea of the model generalization beyond the training dataset. Having a large difference in the two values of loss may introduce a sentiment of overfitting, Data learning, resulting in poor generalization of more recent data.

**Figure 13:**
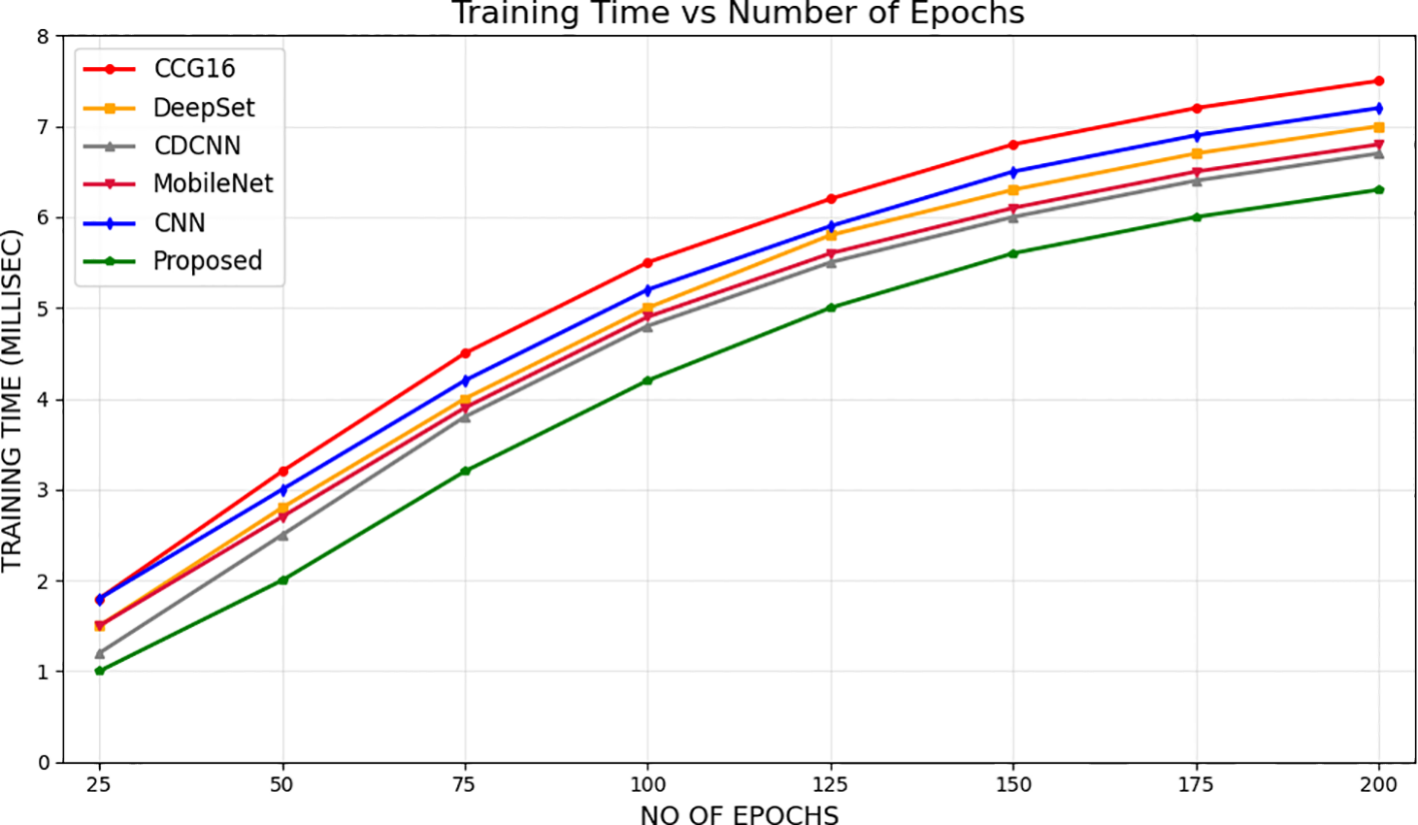
Comparison of computational complexity.

**Figure 14:**
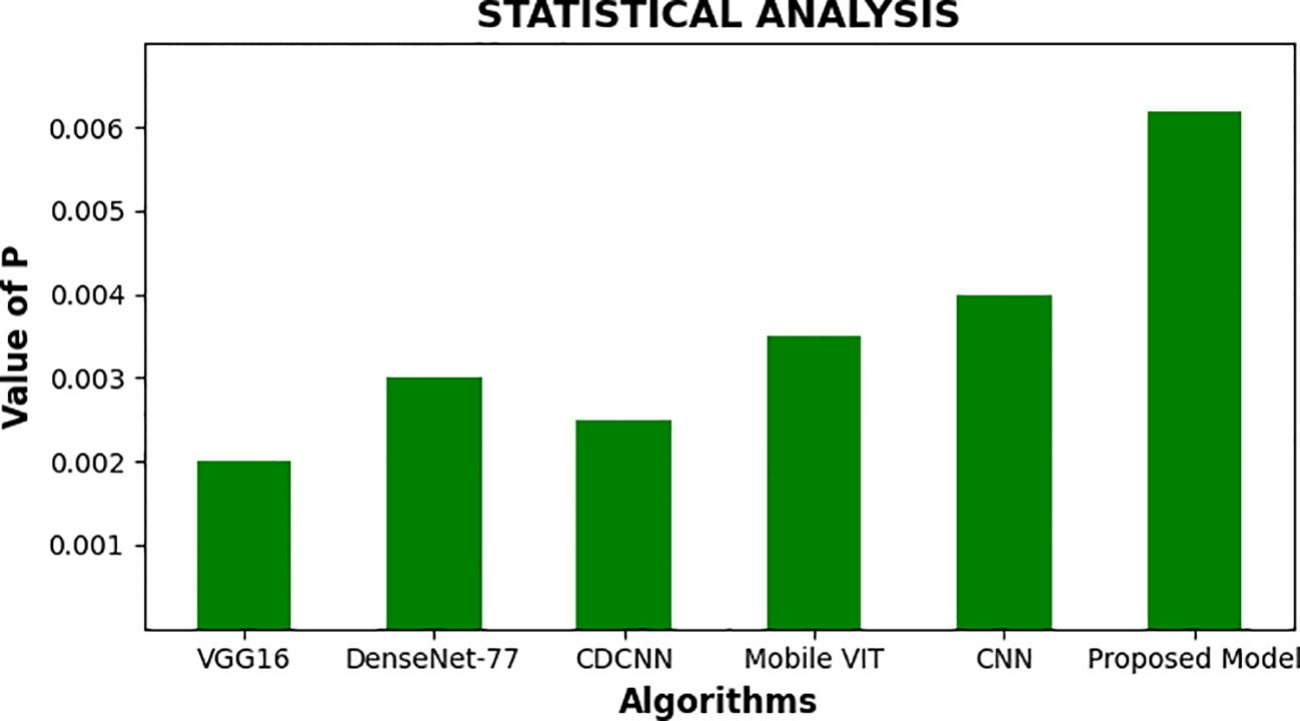
Statistical comparison for the recommended approach among varied optimization approaches.

**Figure 13** provides a comparison of the timely complexity of various approaches to it, considering the processing time, memory consumption, and total resources consumption elements. The analysis plays a significant role in the selection of a good model that will be capable of functioning within the range of limits imposed by the computing infrastructure available with a balance between accuracy and feasibility of implementation. Moreover, **Figure 14** presents a statistical examination of the different optimization techniques, which clearly demonstrates that the suggested algorithm is the best and more reliable in all individual cases. It gives a strong performance in numerous situations, thus ensuring more solid and reliable results.

**Figure 15:**
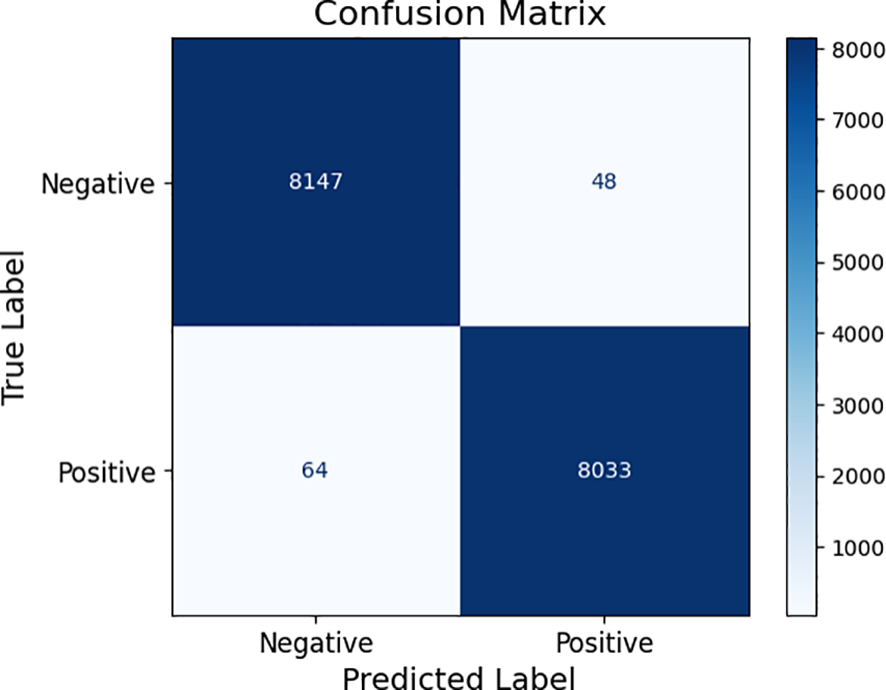
Confusion matrix of the recommended approach in diagnosing plant diseases.

**Table 4** gives an in-depth summary of the performance scores of different optimization algorithms, which are denoted as Algorithms 1 through 5, and compared in terms of their diagnostic performance of different plant diseases on the data of PlantVillage. Parameters, such as precision, accuracy, recall, F1-score, and specificity, are provided to give a refined perception about the effectiveness that each of the methods have when it comes to creating a distinction between diseased and healthy images. **Figure 15** is the visualized model of the confusion matrix of the performance of the proposed approach using PlantVillage dataset. This is a quantitative estimation of correct and incorrect prediction of the model over classes, where accuracy of the classification can be studied in detail.

**Table 5** shows the performance measures of different learning methods that have been tested on a real-time data (supplied by webcams in natural field settings). The real life aspects in the images include lighting differences and noise on the background whereby 10 different classes of 5 different plants are captured. The validation was conducted on almost 200 pictures that were gathered and used. As per **Table 5**, no model is better than the suggested model proving its perfection and ability to be used in real-time scenarios in the agricultural sector.

**Table 5:** Performance metrics of the different optimization approaches using real time dataset.

Algorithms	Accuracy	Precision	Recall	Specificity	F1-score
VGG16	0.795	0.782	0.775	0.758	0.751
DenseNet-77	0.823	0.808	0.829	0.836	0.814
CDCNN	0.812	0.817	0.826	0.813	0.839
Mobile ViT	0.809	0.806	0.824	0.825	0.832
CNN	0.898	0.886	0.872	0.879	0.865
Proposed Model	0.993	0.992	0.993	0.992	0.992

Lastly, **Table 6** illustrates the comparison between our suggested framework and the present systems in the field of plant disease diagnosis. The given information shows the high-accuracy of our method in the field of diagnosing, and it demonstrates its effectiveness as well. As an interesting benefit of our approach, it does not require special hardware to be used, which also makes it both economically and broadly applicable in different user scenarios.

**Table 6:** Comparison of recommended approach with varied existing systems.

Author	Algorithm	Accuracy	Computationally efficient	Specialized hardware requirement
Ref (Kabir et al., 2024)	VGG16 and Darknet53	99.24	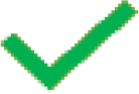	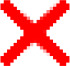
Ref (Albahli and Nawaz, 2022)	DCNet	98.24	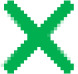	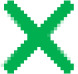
Ref (Saxena and Rathor, 2023)	CNN and Random Forest	–	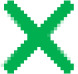	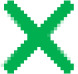
Ref (Barman et al., 2024)	Vision Transformer	90.99	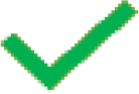	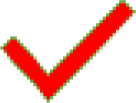
Ref (Kabir et al., 2021)	CNN	–	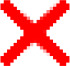	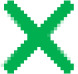
Proposed Model	Enhanced CoAtNets	99.32	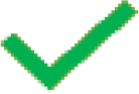	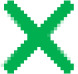

### 4.5 Statistical outcomes and comparison of optimizers

In the process of carrying out our investigation, we have prepared a statistical account of different optimizers, which is depicted in **Table 7**. This comparison analysis is important to analyze the peculiarities of the work of each optimization algorithm in the conditions of the proposed system of plant diseases diagnosis. The **Table 7** summarizes the important measurements like the best, worst, mean, median, standard deviation (SD) and variance of the model met by each optimizer. **Table 8** shows a comparative assessment of MBT model with other baseline models in order to see its diagnostic efficiency with different plant diseases.

**Table 7:** Indicator performance of different optimizers.

Algorithm	Best	Worst	Mean	Median	SD	Variance
VGG16	0.74921	0.64834	0.73011	0.024913	0.068271	5.326 x10^-6^
DenseNet-77	0.72210	0.64390	0.69244	0.019334	0.075434	6.521 x10^-6^
CDCNN	0.75730	0.62680	0.65562	0.034950	0.065551	4.262 x10^-5^
Mobile ViT	0.78955	0.67890	0.71549	0.029243	0.053450	3.135 x10^-4^
CNN	0.84490	0.71512	0.72502	0.032593	0.043532	2.922 x10^-4^
Proposed Model	0.99321	0.81301	0.85741	0.068514	0.039851	1.2895 x10^-4^

**Table 8:** Comparative MBT analysis.

Dataset	VGG 16	DenseNet-77	CDCNN	Mobile ViT	CNN	Proposed Model
Plant Village Dataset	0.50	0.45	0.37	0.39	0.35	0.23

The comparison of the current proposed ensemble heterogeneous transformer model with other commercial models is provided in **Table 9** based on the measurement of multiple plant diseases diagnosis in order to evaluate their efficiency. Through the results, it is apparent that the proposed solution outwits the already existing solution because of its level of accuracy and its applicability within the real-time deployment settings. In the meantime, **Table 10** presents the ablation study, showing the impact of each module on the ensemble model’s performance. Removing U-Net reduced accuracy by 0.9%, and excluding Swin Transformer V2 caused a 1.1% drop, confirming their importance for local and global feature extraction. Excluding CoAtNet reduced accuracy by 2.2%, while removing Enhanced CoAtNet led to the largest drop of 3.1%, showing the value of multiscale feature aggregation and adaptive attention. Eliminating LFHBA fusion reduced accuracy by 1.8%, proving its role in optimizing ensemble predictions. The proposed framework achieved 0.9932 accuracy, confirming that all modules together enhance detection performance. The results of some of the deep learning models (VGG16, DenseNet-77, CDCNN, Mobile ViT, CNN, and the Recommended Model) applied on GPU and CPU are compared on **Table 11**. It is compared based on how long it takes to infer, the amount of memory required, FLOPS, its computational time and the number of parameters to be trained. These results indicate that Recommended method has the lowest inference time and memory consumption rates in the two platforms and has minimum computer requirements. This confirms its efficiency and suitability in real-time application particularly in resources constrained and high-performance computing systems.

**Table 9:** Comparative analysis among commercial and recommended model.

Algorithms	Performance Metrics				
	Accuracy	Precision	Recall	Specificity	F1-score
Google auto ML Vision	0.862	0.849	0.831	0.856	0.823
IBM Watson	0.858	0.831	0.823	0.815	0.814
Microsoft Azure AI	0.892	0.873	0.867	0.852	0.851
Proposed Model	0.993	0.993	0.993	0.993	0.993

**Table 10:** Ablation study for the recommended approach.

Algorithms	Accuracy	Precision	Recall	Specificity	F1-score
Swin Transformer	0.9910	0.9912	0.9913	0.9914	0.9912
U-Net	0.9921	0.9922	0.9923	0.9924	0.9922
CoAtNet	0.9930	0.9931	0.9932	0.9933	0.9934
Enhanced CoAtNet	0.9901	0.9903	0.9902	0.9904	0.9902
Without LFHBA Fusion	0.9914	0.9915	0.9916	0.9916	0.9915
Proposed Model	0.9932	0.9932	0.9931	0.9932	0.9931

**Table 11:** Computational parameters for the different models in both CPU and GPU.

Algorithm	Computational performance (CPU)				
	Inference time (Secs)	Memory (MB)	FLOPS (Secs)	Computational time (Secs)	No. of parameters
Swin Transformer	471.32	18.79	99	77.89	588,891
DenseNet-77	365.01	17.91	99	75.99	458,783
CDCNN	377.91	18.81	99	86.91	442,903
Mobile ViT	120.81	1.20	64	33.79	321,769
CoAtNet	287.51	274.91	99	87.92	487,903
Proposed Model	113.57	1.99	55	26.90	286,891
Swin Transformer	409.45	18.79	99	77.89	588,891
DenseNet-77	351.45	17.91	99	75.99	458,783
CDCNN	255.89	18.83	99	86.91	442,903
Mobile ViT	111.91	1.11	65	33.79	321,769
CoAtNet	233.91	18.31	99	87.92	487,903
Proposed Model	106.79	1.91	56	26.89	286,891

**Figure 16:**
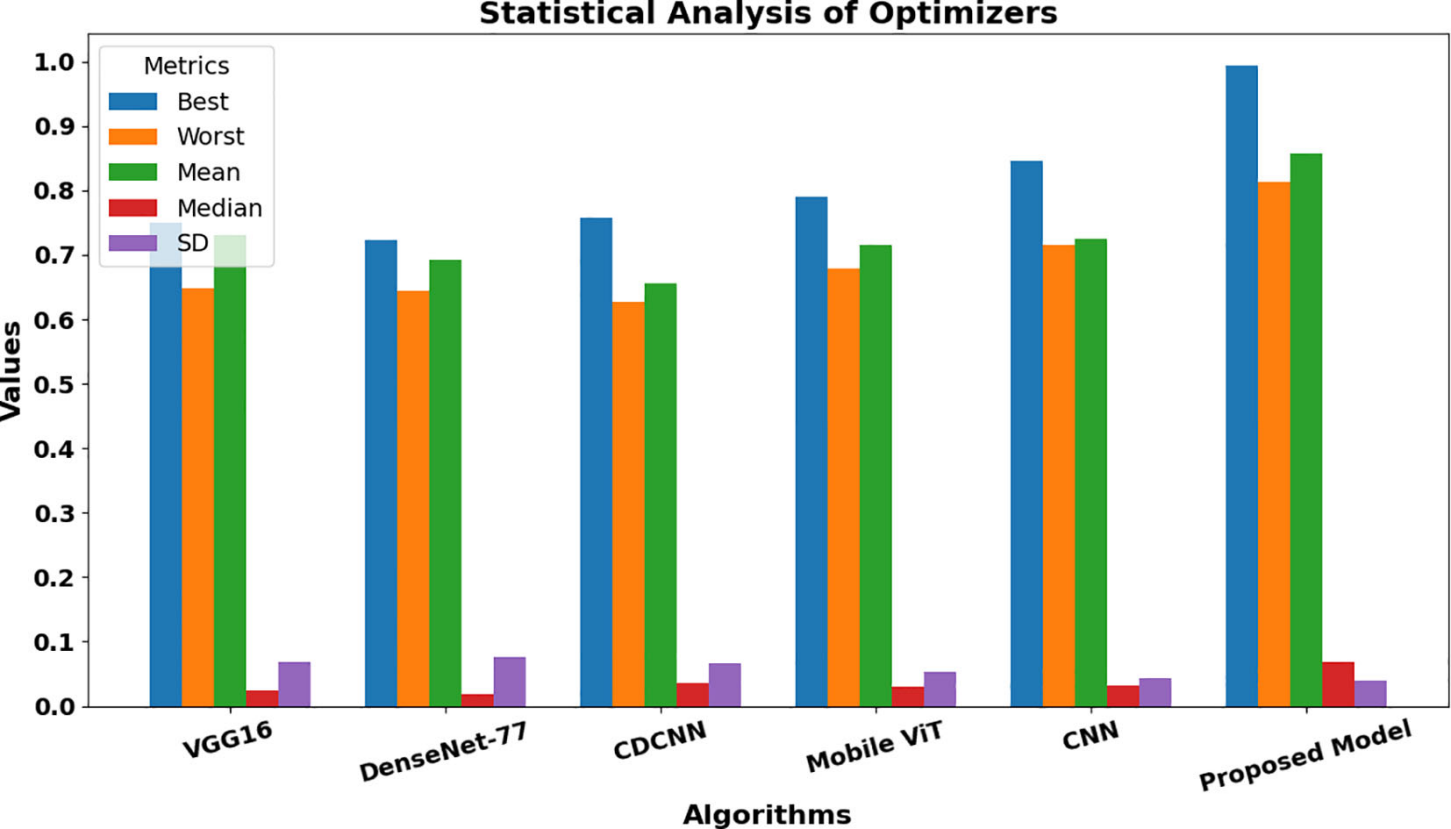
Statistical analysis for different optimizers.

To make these results more graphical, **Figure 16** is presented, which is the statistical analysis of different optimizers provided in the system. **Figure 16** depicts the statistical assessment of distinct optimizers, and reveals their specificities and potential constraints. Further examining the accuracy, the rate of convergence and stability of each of the algorithms, we can gain some idea about the capacity of the algorithms to train the model effectively.

The result of performance analysis of the optimizers is given below:

- VGG16 optimizer achieves the best result in 0.749 and the average measure of 0.72502 is reached at the same time. However, it is a little more fluctuating with variance of 0.043532, which is not good evidence that its performance is not so steady.
- DenseNet-77 is on the other hand not only defined by a low mean accuracy of 0.69244, but also the lower variance of 0.00065 x10–6 suggesting a more stable performance across datasets.
- CDCNN that focuses on spatial information performs with the best result of 0.75730 and the average accuracy of 0.65562. Although it can very well have a variance of 0.00655 x10-5, it is usually slow and unstable.
- Mobile ViT optimizer, a lightweight application optimizer, uses a mean of 0.71549 with a commendable maximum value of 0.78955. However, its variance is more at 0.003135 x10–4 meaning that it might not be as steady as the other optimizers in some situations.

The CNN, which is standard, obtains a mean accuracy of 0.72502 and a standard deviation of 0.002922 x10–4 which indicates its popularity in the domain of computer vision.

- The Mobile ViT optimizer, designed for lightweight applications, demonstrates a mean accuracy of 0.71549, with a noteworthy best value of 0.78955. Its variance, however, is higher at 0.003135 x10^-4, indicating that it may not be as consistent as other optimizers in certain scenarios.
- The CNN, a classic approach, achieves a mean accuracy of 0.72502 with a variance of 0.002922 x10^-4, reflecting its well-established status in the field of computer vision.
- Our proposed model, incorporating a sophisticated blend of U-Net, Swin Transformer V2, and TNNs such as CoAtNets and Enhanced CoAtNets, outshines the others with a mean accuracy of 0.85741, a best performance of 0.9932, and the lowest variance of 0.0012895 x10^-4. This underscores the efficacy of our approach in balancing high accuracy with reliable performance.

The statistical analysis presented here provides a quantitative foundation for understanding the optimizer landscape and guiding the selection of the most appropriate one for the specific needs of our plant disease diagnosis system. The convergence speed and stability are particularly significant, as they directly impact the approach’s capability to generalize and adapt to new, unseen data, which is critical in real-world agricultural settings.

### 4.6 Interpretability analysis

**Figure 17:**
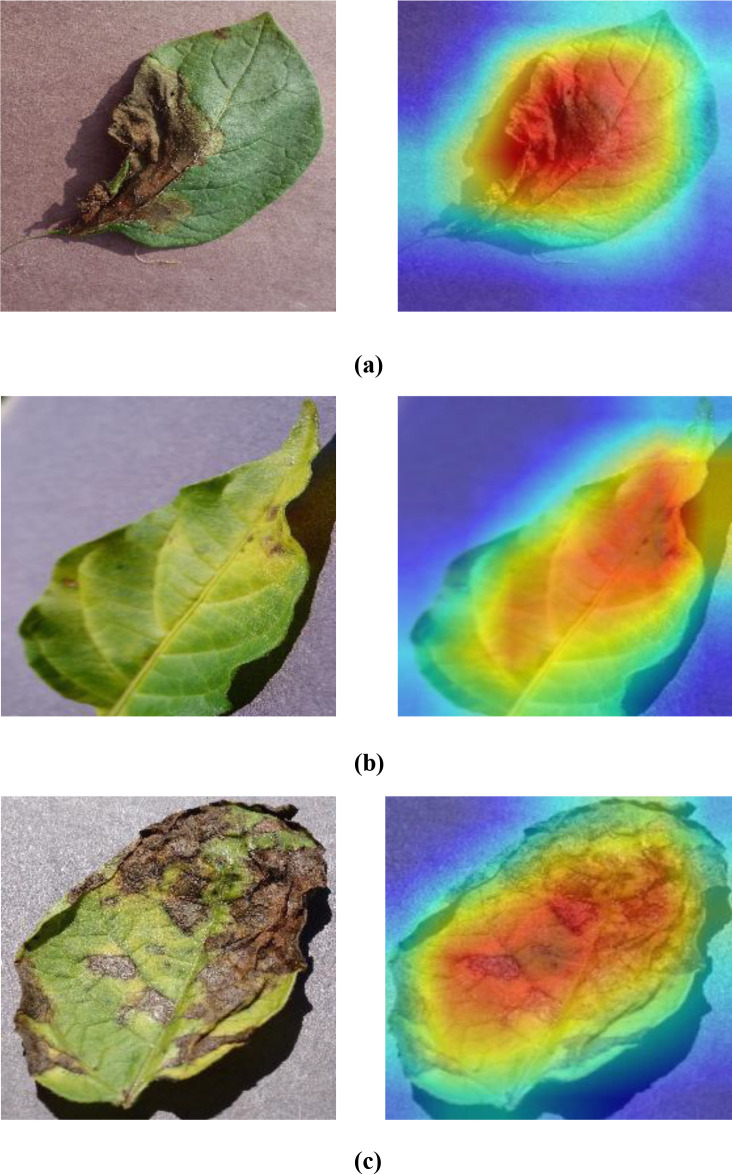
**(a–c)** Interpretability analysis using GRAD-CAM for plant disease localization.

In this segment, the GRAD-CAM method is adopted to map regions of disease in plant leaves across the PlantVillage dataset. **Figures 17a-c** of GRAD-CAM visualizations provide the original images of leaves and their respective heatmaps, which are useful in identifying the most important areas of the leaf that would aid in disease classification. The presentation shows that the model is able to recognize the symptoms of a disease with accuracy, as well as gives us an idea of how it sees spatial features, hence improving the transparency and interpretability of the detection process.

Overall, the obtained results confirm the efficiency, scalability, and interpretability of the ensemble heterogeneous transformer model. The subsequent section discusses the broader implications and possible enhancements of this framework in future agricultural systems.

## 5 Conclusion and future directions

The research paper ends up with the proposal of a novel and extremely effective heterogeneous ensemble deep learning system to easily and accurately detect plant diseases. This framework combines the superior methods of image segmentation including U-Net and Swin Transformer V2 with the power of Transformer Neural Networks including CoAtNet and Enhanced CoAtNet. The two-step method increases the accuracy of the segmentation and disease classifications. The framework is further enhanced with a meta-heuristic decision fusion strategy which is used to combine the results of separate models. In order to be more transparent and explainable, the GRAD-CAM technique is used to create heat maps of the leaf areas that have the largest impact on the model outputs. The empirical results are convincing with 7.34% better than single-method selection, 8.43% better than homogeneous ensemble models, and a massive 14.59% better than the single deep learning models. In addition, it shows high performance in motor onset category. In general, the combination of heterogeneous architectures and complementary advantages provides an important innovation in plant pathology, as a platform to a sustainable agriculture and world food security. Even though in the current work security issues are not being considered, the extensions of the work in the future will include secure data treatment and on-device processing to eliminate the risks of privacy. Data protection laws such as GDPR will also be addressed as everyone would like to have ethical data deployment in the IoT-based and mobile-based agricultural systems. Moreover, SHAP or LIME can be used together with GRAD-CAM in future research to improve interpretability.

## Data Availability

The original contributions presented in the study are included in the article/supplementary material. Further inquiries can be directed to the corresponding authors.
